# Epstein-Barr virus BNRF1 destabilizes SMC5/6 cohesin complexes to evade its restriction of replication compartments

**DOI:** 10.1016/j.celrep.2022.110411

**Published:** 2022-03-08

**Authors:** Stephanie Pei Tung Yiu, Rui Guo, Cassie Zerbe, Michael P. Weekes, Benjamin E. Gewurz

**Affiliations:** 1 Division of Infectious Diseases, Department of Medicine, Brigham and Women’s Hospital, 181 Longwood Avenue, Boston, MA 02115, USA; 2 Harvard Graduate Program in Virology, Boston, MA 02115, USA; 3 Broad Institute of Harvard and MIT, Cambridge, MA 02142, USA; 4 Department of Microbiology, Harvard Medical School, Boston, MA 02115, USA; 5 Cambridge Institute for Medical Research, University of Cambridge, Hills Road, Cambridge CB2 0XY, UK; 6 Lead contact

## Abstract

Epstein-Barr virus (EBV) persistently infects people worldwide. Delivery of ~170-kb EBV genomes to nuclei and use of nuclear membrane-less replication compartments (RCs) for their lytic cycle amplification necessitate evasion of intrinsic antiviral responses. Proteomics analysis indicates that, upon B cell infection or lytic reactivation, EBV depletes the cohesin SMC5/6, which has major roles in chromosome maintenance and DNA damage repair. The major tegument protein BNRF1 targets SMC5/6 complexes by a ubiquitin proteasome pathway dependent on calpain proteolysis and Cullin-7. In the absence of BNRF1, SMC5/6 associates with R-loop structures, including at the viral lytic origin of replication, and interferes with RC formation and encapsidation. CRISPR analysis identifies RC restriction roles of SMC5/6 components involved in DNA entrapment and SUMOylation. Our study highlights SMC5/6 as an intrinsic immune sensor and restriction factor for a human herpesvirus RC and has implications for the pathogenesis of EBV-associated cancers.

## In brief

Yiu et al. identify that Epstein-Barr virus subverts the SMC5/6 cohesin complex upon B cell infection and lytic reactivation. The viral tegument protein BNRF1 targets SMC5/6 for proteasomal degradation via Cullin 7 and calpain. Without BNRF1, SMC5/6 can sense viral genomic R-loops and prevent formation of lytic replication compartments.

## Graphical Abstract

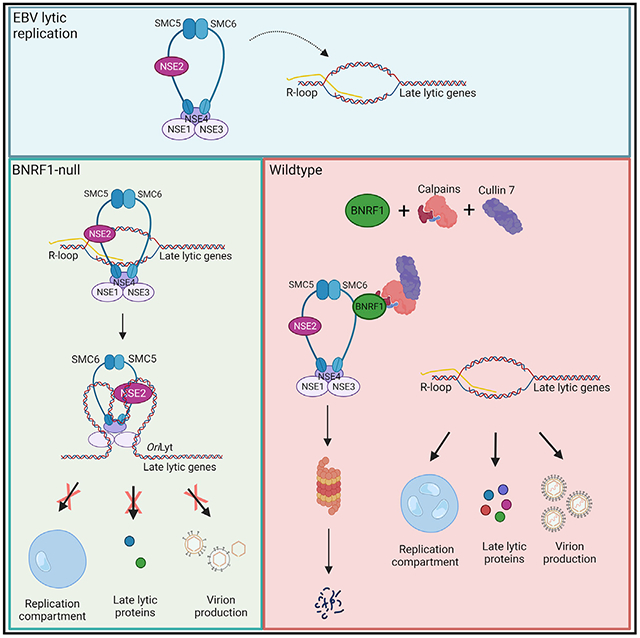

## INTRODUCTION

Epstein-Barr virus (EBV) establishes life-long infection in more than 95% of adults worldwide, is the etiologic agent of infectious mononucleosis, and is associated with multiple sclerosis and with ~1% of human cancers (Parkin, 2006; Zur Hausen and de Villiers, 2015). These include endemic Burkitt lymphoma, Hodgkin’s lymphoma, post-transplantation and HIV-associated lymphoma, T and natural killer (NK) cell lymphomas, and nasopharyngeal and gastric carcinomas (Farrell, 2019; Shannon-Lowe et al., 2017). Much remains to be learned about how EBV subverts host immune barriers to establish latency, reactivate within the heart of the adaptive immune system, and cause cancer.

The Epstein-Barr virion is comprised of a 170-kb double-stranded DNA (dsDNA) genome packaged in an icosahedral capsid that is surrounded by a proteinaceous tegument and lipid envelope (Rixon and Schmid, 2014). Upon host cell infection, tegument proteins are released, and the EBV capsid traffics to the nuclear pore, where viral genomes are inserted into the nucleus, chromatinized, and circularized. The EBV tegument protein BNRF1 disrupts ATRX/DAXX complexes to prevent loading of repressive H3.3 histones onto incoming EBV genomes (Tsai et al., 2011). Knowledge remains incomplete about how EBV evades foreign DNA sensors in newly infected cells (Buschle and Hammerschmidt, 2020; Chakravorty et al., 2019; Lieberman, 2013).

Upon EBV lytic reactivation, the immediate-early genes BZLF1 (also called ZTA or Zebra) and BRLF1 (also called RTA) induce 32 viral early genes that initiate lytic EBV genome synthesis (Kenney and Mertz, 2014; Miller and El-Guindy, 2002). EBV lytic genes form membrane-less nuclear replication compartments (RCs), in which the EBV-encoded polymerase BALF5 produces new genomes. EBV RCs occupy nearly 30% of nuclear volume, which itself is doubled upon lytic reactivation (Nagaraju et al., 2019; Speck et al., 1997). The polymerase processivity factor BMRF1 is found exclusively within RCs, where hundreds of newly synthesized copies of EBV DNA are organized around BMRF1 cores (Daikoku et al., 2005; Nagaraju et al., 2019; Sugimoto et al., 2013). Whether these structures can be sensed by innate immune sensors is unknown. Two EBV origins of lytic DNA replication (*oriLyts*) serve as key *cis*-acting enhancers of late lytic gene expression (Djavadian et al., 2016; Hammerschmidt and Sugden, 2013). GC-rich regions form RNA:DNA hybrid R loop structures at both *oriLyts* (Rennekamp and Lieberman, 2011).

Approximately 30 EBV late genes are transcribed from newly synthesized lytic EBV genomes and encode virion capsid, tegument, and glycoproteins. It is not completely understood why EBV late genes require production of nascent DNA in RCs, but *oriLyts* serve key *cis*-acting enhancer roles in late gene expression (Djavadian et al., 2016). Ongoing EBV DNA replication maintains RC integrity (Li et al., 2018).

The structural maintenance of chromosomes (SMC) condensin, cohesin, and SMC5/6 are ATP-powered, ring-shaped machines that topologically entrap DNA and are major regulators of DNA replication, transcription, and chromosome biology (Uhlmann, 2016). Recent studies highlight the capability of SMC5/6 to repress transcription from dsDNA viral genomes in the absence of viral evasion mechanisms (Bentley et al., 2018; Decorsiere et al., 2016; Dupont et al., 2021; Gibson and Androphy, 2020; Murphy et al., 2016; Niu et al., 2017; Xu et al., 2018). To avoid silencing, the hepatitis B virus (HBV) HbX oncoprotein assembles a ubiquitin ligase complex to target SMC6 for proteasomal degradation (Decorsiere et al., 2016; Murphy et al., 2016; Niu et al., 2017). In the absence of HbX, SMC5/6 interacts with the episome to inhibit viral transcription, but the mechanism by which it recognizes viral DNA and alters its expression remains incompletely understood. Similarly, adenovirus-encoded E4 targets SMC5/6 for degradation, in the absence of which SMC5/6 localizes to viral RCs, associates with replicating adenoviral dsDNA genomes, and impairs viral lytic DNA replication (Dybas et al., 2021). Much remains to be learned about how SMC5/6 recognizes double-stranded viral DNA and whether it can recognize herpesvirus genomes, including that of EBV, in newly infected or lytic cells.

Here we used recently constructed temporal proteomic maps (Ersing et al., 2017; Wang et al., 2019a) to identify that SMC5/6 is depleted upon primary human EBV B cell infection and again upon B or epithelial cell EBV lytic reactivation. We identify BNRF1 as necessary and sufficient for SMC5/6 depletion and determine that it mediates SMC5/6 degradation in a calpain-, Cullin-7 ubiquitin ligase-, and proteasome-dependent manner. In the absence of BNRF1, SMC5/6 interacts with RNA:DNA hybrid R-loop structures, including at *oriLyts* to suppress EBV RC formation, genome encapsidation, sustained late gene expression, and infectious virion production. These studies implicate SMC5/6 as a key host restriction factor for a herpesvirus RC.

## RESULTS

### BNRF1 meditates SMC5/6 complex turnover in EBV B and epithelial cell lytic replication

**Figure 1.**
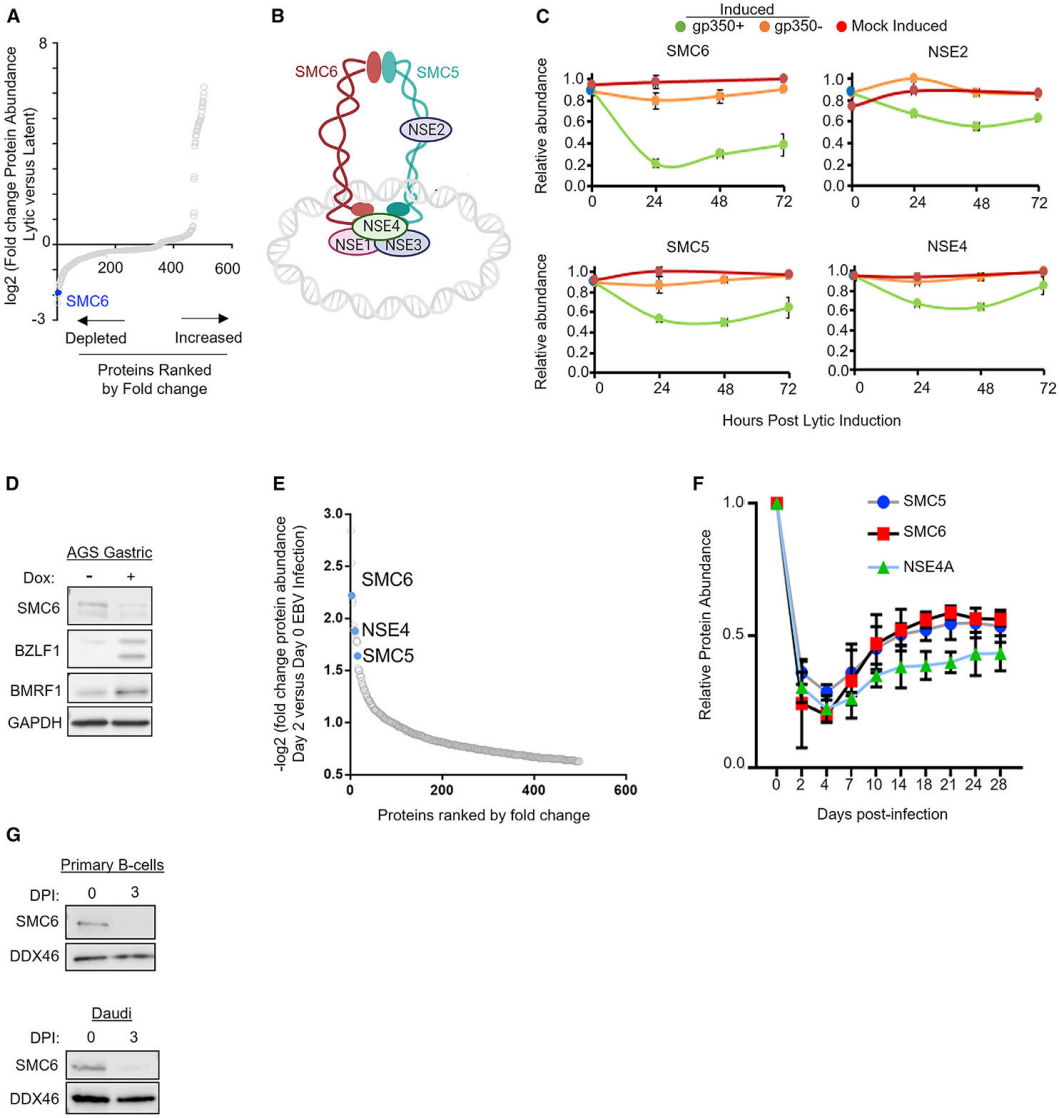
The SMC5/6 cohesin complex is depleted by incoming EBV and lytic replication (A) Waterfall plot illustrating the log2 fold change in protein abundance of host proteins that significantly change (p < 0.05) 24 h after lytic reactivation of P3HR-1 ZHT/RHT cells mock-induced or induced for lytic reactivation by 4-HT (Ersing et al., 2017). (B) Schematic of the SMC5/6 complex. (C) Temporal proteomic plots of SMC5/6 complex subunit relative protein abundances in mock-induced (red) or 4-HT induced ZHT/RHT P3HR-1 cells that entered productive lytic cycles and expressed the late gene gp350 (green), or that did not become gp350+ (orange), are indicated. Error bars indicate mean ± SE (n = 3 replicates). (D)Immunoblot analysis of whole-cell lysates (WCLs) from EBV+ AGS gastric carcinoma cells with a conditional BZLF1 allele induced for lytic replication by doxycycline (2 μg/mL) for 48 h. (E) Plot of −log2 fold change in protein abundance of host proteins that were significantly different in proteomics analysis of primary human B cells infected with B95.8 EBV at 48 h of infection versus in resting B cells. (F) Temporal proteomics plots of SMC5/6 complex abundance on the indicated days after primary human B-cell EBV infection. Data show the mean ± SE (n = 4). (G) Immunoblot of SMC6 versus the DDX46 load control values in primary (top) or Daudi B cells at 0 versus 3 days post infection (DPI). Blots in (D) and (G) are representative of 2 replicates. See also Figure S1.

To identify how EBV lytic replication remodels the B cell proteome, we recently used whole-cell, tandem mass tag-based analysis to generate unbiased temporal profiles of nearly 8,000 host and 69 viral proteins in two Burkitt lymphoma B cell lines induced for lytic reactivation (Ersing et al., 2017). For this proteomic analysis, lytic reactivation was triggered in P3HR-1 cells engineered to stably express conditional EBV immediate early ZTA and RTA alleles fused to a 4-hydroxy tamoxifen (4-HT)-dependent mutant estrogen receptor binding domain (ZHT and RHT, respectively). 4-HT addition induces ZHT/RHT nuclear translocation and triggers lytic replication in P3HR-1 ZHT/RHT cells (Calderwood et al., 2008). As a complementary approach, lytic reactivation was also triggered in Akata cells by immunoglobulin receptor cross-linking (Ersing et al., 2017). Interestingly, re-analysis of the proteomic dataset identified that multiple components of the SMC5/6 complex were rapidly depleted in P3HR-1 and Akata cells, which harbor type I versus II EBV strains, respectively. SMC6 was among the most highly depleted human protein within 24 h of lytic reactivation (Figures 1A-1C), raising the question of whether it can restrict EBV lytic replication in the absence of viral subversion. EBV is associated with 10% of gastric carcinomas, and we similarly observed that SMC6 abundance was reduced during EBV lytic reactivation in EBV+ AGS gastric carcinoma cells with a doxycycline-inducible immediate-early ZTA allele (Verma et al., 2016; Figure 1D).

We next asked whether SMC5/6 complex abundance was perturbed during EBV infection of primary human B cells, which results in latency as opposed to lytic replication. Using our temporal proteomic map (Wang et al., 2019a), we again found that multiple SMC5/6 cohesin complex subunits were among the most highly depleted human proteins 48 h after EBV infection (Figure 1E). The abundance of SMC5, SMC6, and NSE4A cohesin reached a nadir on day 4 after infection, a time point when cells begin to rapidly proliferate (Nikitin et al., 2010; Figures 1F and S1A). However, SMC6 mRNA increased over the first 48 h after infection, suggesting that changes in its protein abundance likely occurred at the post-transcriptional level. At later time points, the abundance of SMC5, SMC6, and NSE4A mRNAs decreased, perhaps indicative of a second mechanism by which EBV suppresses SMC5/6 following latency establishment (Figure S1A). We validated SMC6 loss on day 3 after EBV infection of primary and Burkitt B cells (Figures 1G, S1B, and S1C).

### The EBV major tegument protein BNRF1 targets SMC5/6 for proteasomal degradation

**Figure 2.**
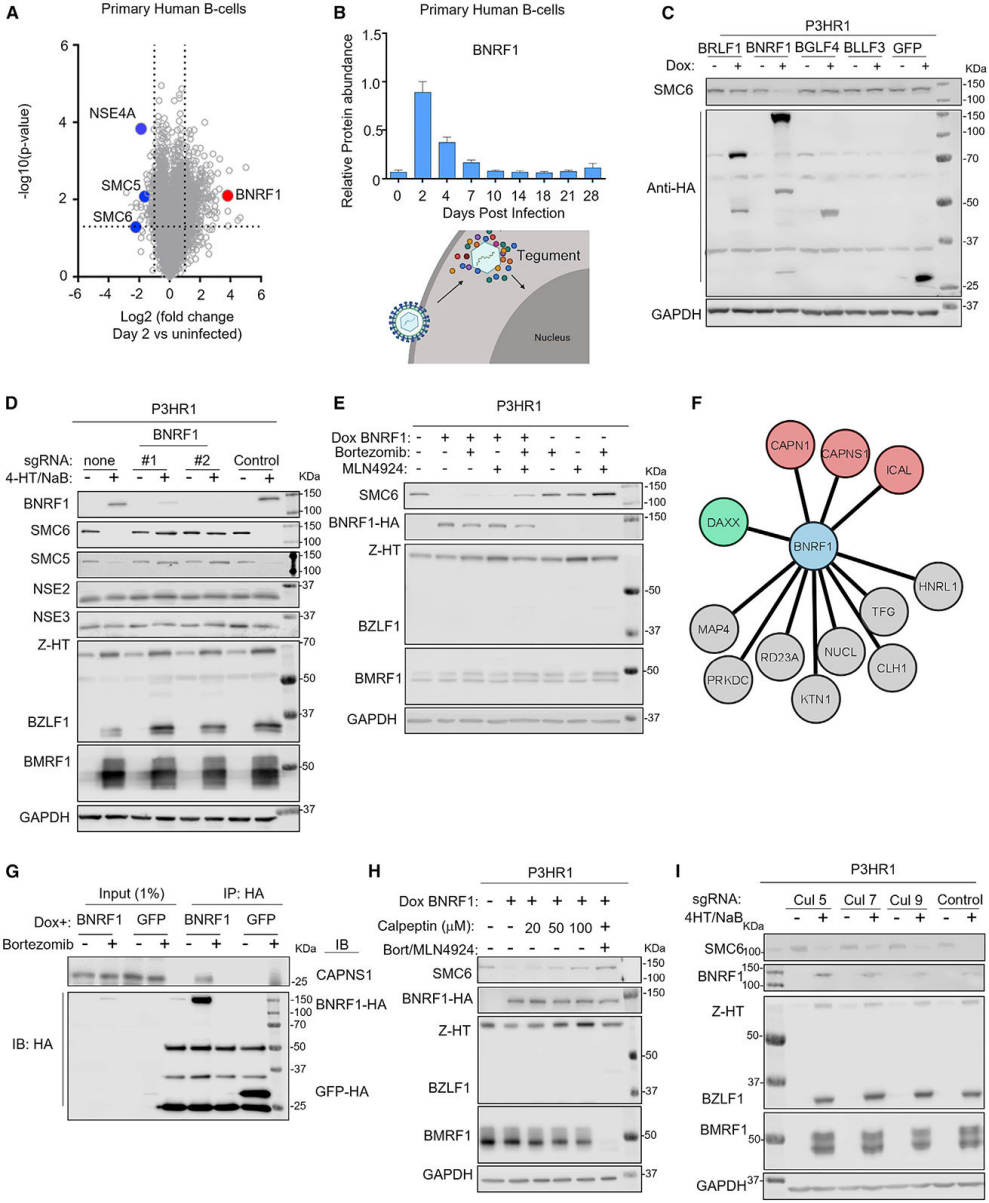
The EBV tegument protein BNRF1 targets SMC5/6 for proteasomal degradation in a calpain-dependent manner (A) Volcano plot analysis of changes in proteomic abundances in primary human B cells at 2 DPI versus uninfected. (B) Relative BNRF1 protein abundances at the indicated days after primary B cell EBV infection. (C) Immunoblot analysis of WCL from P3HR-1 cells mock induced or doxycycline (dox; 5 μg/mL) induced for EBV lytic gene cDNA expression for 24 h. (D) Immunoblot analysis of WCL from Cas9+ P3HR-1 ZHT/RHT cells expressing the indicated sgRNA and mock induced or induced for lytic reactivation by 4-HT (400 nM) and sodium butyrate (NaB; 500 μM) for 24 h. (E) Immunoblot analysis of P3HR-1 ZHT/RHT WCL from cells mock induced or dox induced for BNRF1 cDNA expression in the presence of bortezomib (5 nM) or MLN4924 (10 μM), as indicated, for 24 h. (F) BNRF1-selective protein interactors identified by affinity purification, HA peptide elution, and mass spectrometry analysis of dox-induced HA-BNRF1 in P3HR-1 ZHT/RHT cells induced for lytic replication by 4-HT/NaB and cross-compared with the HA-tagged tegument protein controls BPLF1, BLRF2 and BOLF1. (G) Immunoblot analysis of 1% input and anti-HA immunopurified GFP or SMC6 complexes from P3HR-1 ZHT/RHT left untreated or treated with dox (5 μg/mL) and bortezomib (5 nM) for 6 h, as indicated. Representative of two independent experiments. (H) Immunoblot analysis of WCL from P3HR-1 ZHT/RHT cells with dox-induced HA-tagged BNRF1 and treated with bortezomib, MLN4924, or calpeptin for 24 h, as indicated. (I) Immunoblot analysis of WCL from P3HR-1 ZHT/RHT cells expressing the indicated sgRNAs and induced for lytic replication, as indicated. Blots are representative of 2 independent replicates unless otherwise indicated. See also Figure S2.

Proteomic analysis detected multiple EBV capsid, tegument, and glycoproteins over the first 96 h post infection (hpi), likely delivered by incoming viral particles because most early gene products that do not encode virion components were not detected (Wang et al., 2019a). The EBV major tegument protein BNRF1 was among the most abundant EBV protein at 48 hpi (Figure 2A), where it was maximally expressed (Figure 2B). To test whether BNRF1 was sufficient to mediate SMC6 depletion, a panel of P3HR-1 B cells was established with conditional hemagglutinin (HA) epitope-tagged EBV lytic protein expression. Doxycycline induction of BNRF1, but not of the other EBV lytic proteins or a GFP control, resulted in SMC6 loss (Figure 2C). To avoid possible confounding effects on expression from latent P3HR-1 EBV genomes, EBV- BJAB B cells with conditional BNRF1 expression were established. BNRF1 expression was sufficient for SMC6 depletion (Figure S2A).

We next wanted to determine whether BNRF1 was necessary for SMC6 loss upon EBV lytic reactivation. For this analysis, we used Cas9+ P3HR-1 ZHT/RHT (Calderwood et al., 2008) triggered for EBV lytic reactivation by addition of 4-HT and the histone deacetylase inhibitor sodium butyrate (NaB). To establish CRISPR-edited cells, control or two independent single guide RNAs (sgRNAs) against *BNRF1* were expressed. 4-HT and NaB addition induced expression of endogenous immediate-early BZLF1 and early BMRF1 genes in control and *BNRF1*-edited cells. However, SMC5/6 depletion was strongly impaired by BNRF1 knockout (KO) (Figure 2D). Levels of the NSE2 and NSE3 SMC5/6 cohesin components were not diminished, indicating that DNA-bound cohesin subunits are preferentially targeted (Figures 1B and 2D).

Rapid SMC5/6 loss raised the possibility that BNRF1 targets the SMC5/6 complex for degradation. In support of this, immunoblot analysis revealed that SMC6 levels were already strongly reduced by 15 hpi (Figure S2B). The proteasome inhibitor bortezomib or the small molecule neddylation antagonist MLN4924, which blocks activity of Cullin-based ubiquitin E3 ligases, each diminished SMC6 depletion upon inducible BNRF1 expression. Co-administration of bortezomib and MLN4924 had additive effects (Figure 2E), indicating that BNRF1 likely utilizes a Cullin ubiquitin ligase to target SMC5/6 for proteasomal degradation.

BNRF1 is not known to have ubiquitin ligase activity. Therefore, to gain insights into host proteins used by BNRF1 to deplete SMC5/6, HA-tagged BNRF1 or the control EBV tegument proteins BPLF1, BLRF2, and BOLF1 were inducibly expressed and purified by HA peptide elution from P3HR-1 ZHT/RHT cells triggered for lytic replication. High-confidence BNRF1-selective interactors were identified by liquid chromatography-mass spectrometry analysis of immunopurified material, followed by Comparative Proteomic Analysis Software Suite (CompPASS) analysis (Sowa et al., 2009; Huttlin et al., 2015). This analysis identified DAXX as a key BNRF1 interactor, in keeping with prior published studies (Huang et al., 2016; Tsai et al., 2011). SMC6 was not detected, perhaps reflecting transient association with BNRF1 in the absence of Cullin or proteasome inhibitors. Unexpectedly, multiple calpain subunits were high-confidence BNRF1 interactors, including the catalytic CAPN1 and regulatory CAPNS1 subunits (Figures 2F and S2C). Calpains are calcium-dependent cysteine proteases that cleave particular protein substrates to facilitate proteolytic processes (Ono and Sorimachi, 2012). We validated that the calpain proteolytic subunit CAPNS1 co-immunoprecipitated with inducibly expressed BNRF1 in bortezomib-treated cells and also with SMC6 in cells induced for lytic replication (Figures 2G and S2D).

To test whether calpain enzymatic activity was necessary for SMC6 depletion, BNRF1 was induced in the absence or presence of the highly selective calpain inhibitor calpeptin. A dose-response relationship was observed, and calpeptin rescued SMC6 expression to nearly the same extent as bortezomib/MLN4924 treatment in P3HR-1 cells induced for BNRF1 cDNA expression (Figure 2H). Interestingly, calpain inhibition by *CAPNS1* KO or calpeptin rescued SMC6 expression upon lytic replication (Figures S2E and S2F). Calpain inhibition also reduced expression of BNRF1, but not of the early gene BMRF1 (Figures S2E and S2F). Given that calpeptin did not alter levels of conditionally expressed BNRF1, this result raises the possibility that SMC6 stabilization down-modulates EBV late gene expression.

Calpain proteolysis can generate proteolytic fragments that are then subjected to ubiquitin-dependent degradation by the Arg/N-end rule pathway, which uses Cullin E3 ligases (Piatkov et al., 2014; Varshavsky, 2019). We therefore tested whether CRISPR KO of six Cullin genes or the DDB1 adaptor protein of Cullin 4A/B complexes could stabilize SMC6 in P3HR-1 cells induced for lytic replication. Although it has not been associated previously with the N-end rule pathway, Cul7 KO stabilized SMC6 (Figures 2I and S2G). Cul7, but not Cul1, Cul3, or DDB1, co-immunoprecipitated with BNRF1 in lysates from bortezomib-treated P3HR-1 cells induced for lytic replication (Figure S2H). SMC6 complexes immunopurified from whole-cell lysates of P3HR-1 induced for lytic replication were highly modified by high-molecular-weight polyubiquitin chains, as judged by immunoblot analysis with the anti-ubiquitin antibody P4D1 (Figure S2I). These data support a model where BNRF1 drives calpain- and Cul7-dependent SMC6 turnover.

### BNRF1 and SMC6 associate at nuclear puncta

**Figure 3.**
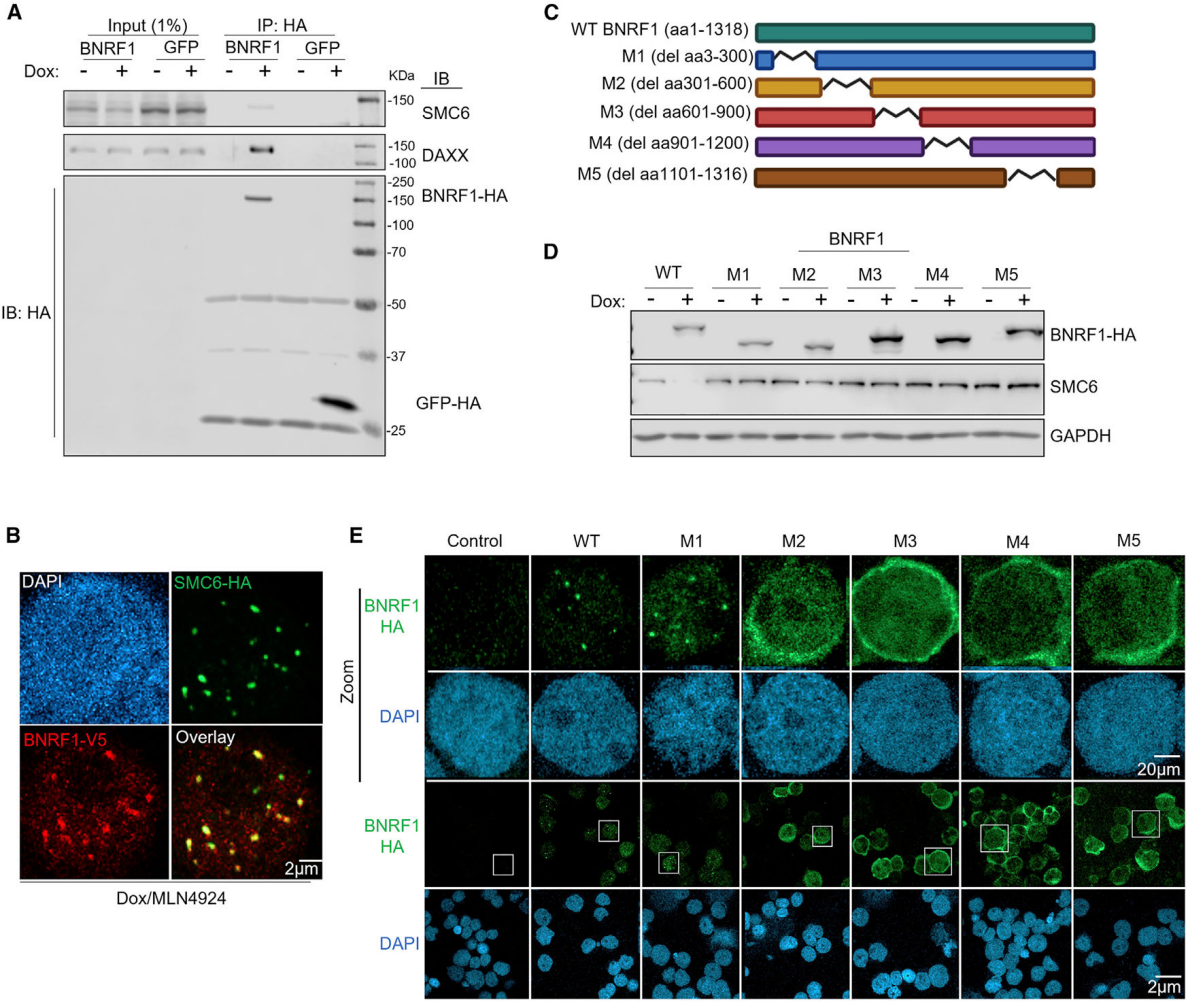
BNRF1 associates with SMC6 (A) Immunoblot analysis of 1% input and anti-HA immunopurified complexes from P3HR-1 ZHT/RHT left untreated or induced for GFP versus BNRF1 cDNAs with dox (5 μg/mL) for 6 h and treated with bortezomib (5 nM) for 6 h. Representative of two independent experiments. (B) Immunofluorescence analysis of dox-induced (5 μg/mL) HA-tagged SMC6 and V5-tagged BNRF1 versus nuclear DAPI signals in EBV+ Akata cells treated with bortezomib (5 nM) and MLN4924 (10 μM) for 12 h. Scale bar, 2 μm. Representative of 3 experiments. (C) Schematic of BNRF1 wild-type (WT) and deletion mutant constructs used. (D) Immunoblot analysis of WCL from P3HR-1 ZHT/RHT cells left untreated or induced for BNRF1 cDNA expression by dox (5 μg/mL) for 24 h. Representative of 2 replicates. (E) Immunofluorescence analysis of dox-induced (5 μg/mL) HA-tagged BNRF1 expression for 24 h versus DAPI staining in P3HR-1 ZHT/RHT cells. Magnified images of cells boxed in white are shown in the top two rows. Representative of 3 experiments. See also Figure S3.

BNRF1 binds the histone chaperone DAXX to disrupt ATRX/DAXX complexes and prevent loading of repressive histone 3.3 onto incoming EBV genomes in newly infected cells (Tsai et al., 2011). We wanted to determine whether BNRF1 likewise associates with SMC5/6 cohesin complexes by two approaches. First, HA-epitope tagged BNRF1 was inducibly expressed in P3HR-1 ZHT/RHT cells in the presence of bortezomib, immunopurified, and subjected to SDS-PAGE. SMC6 as well as DAXX co-immunoprecipitated with BNRF1 (Figure 3A). Stably expressed HA-SMC6 reciprocally co-immunoprecipitated endogenous BNRF1 from bortezomib-treated lytic P3HR-1 ZHT/RHT (Figure S3A). Second, inducibly expressed HA-SMC6 and stably over-expressed V5-BNRF1 co-localized in EBV+ Akata B cell nuclear puncta in MLN4924-treated cells (Figure 3B). Intriguingly, the subnuclear distribution of stably overexpressed BNRF1 changed substantially in the presence of conditionally expressed SMC6, from a diffuse nuclear pattern to puncta that highly overlapped that of SMC6 (Figure S3B). 3D image reconstruction showed that these puncta were nuclear (Figures S3C and S3D). Presumably, stable BNRF1 expression achieved levels in excess of endogenous SMC6, and co-expression of HA-SMC6 provided sufficient substrate to re-localize BNRF1 into nuclear SMC6 foci.

BNRF1 residues 301–600 form a globular domain that mediates association with histone H3.3/H4-bound DAXX at promyelocytic leukemia (PML) nuclear bodies (Huang et al., 2016; Tsai et al., 2011). This interaction leads to disruption of the ATRX/DAXX complex without targeting it for degradation. Because association with BNRF1 instead results in a different fate for the SMC5/6 cohesin complex, we characterized BNRF1 regions that interact with SMC6. BNRF1 wild type versus internal deletion mutants, termed M1-M5, were inducibly expressed in P3HR-1 ZHT/RHT (Figure 3C). Surprisingly, induction of wild-type (WT) but not deletion mutant BNRF1 caused SMC6 depletion, even though M1–M5 were expressed at similar or greater levels than WT BNRF1 (Figure 3D). In agreement with prior studies, co-immunoprecipitation analysis revealed BNRF1 residues 301–600 to be essential for association with DAXX. However, each BNRF1 region, particularly M5, was important for association with SMC6 (Figure S3E). Unexpectedly, we found that M2–M5 failed to form nuclear puncta and instead exhibited perinuclear distribution (Figure 3E). These results suggest that multiple BNRF1 regions are likely important for association with SMC6 and localization to nuclear puncta.

### Key BNRF1 roles in lytic DNA replication, late gene expression, and morphogenesis

**Figure 4.**
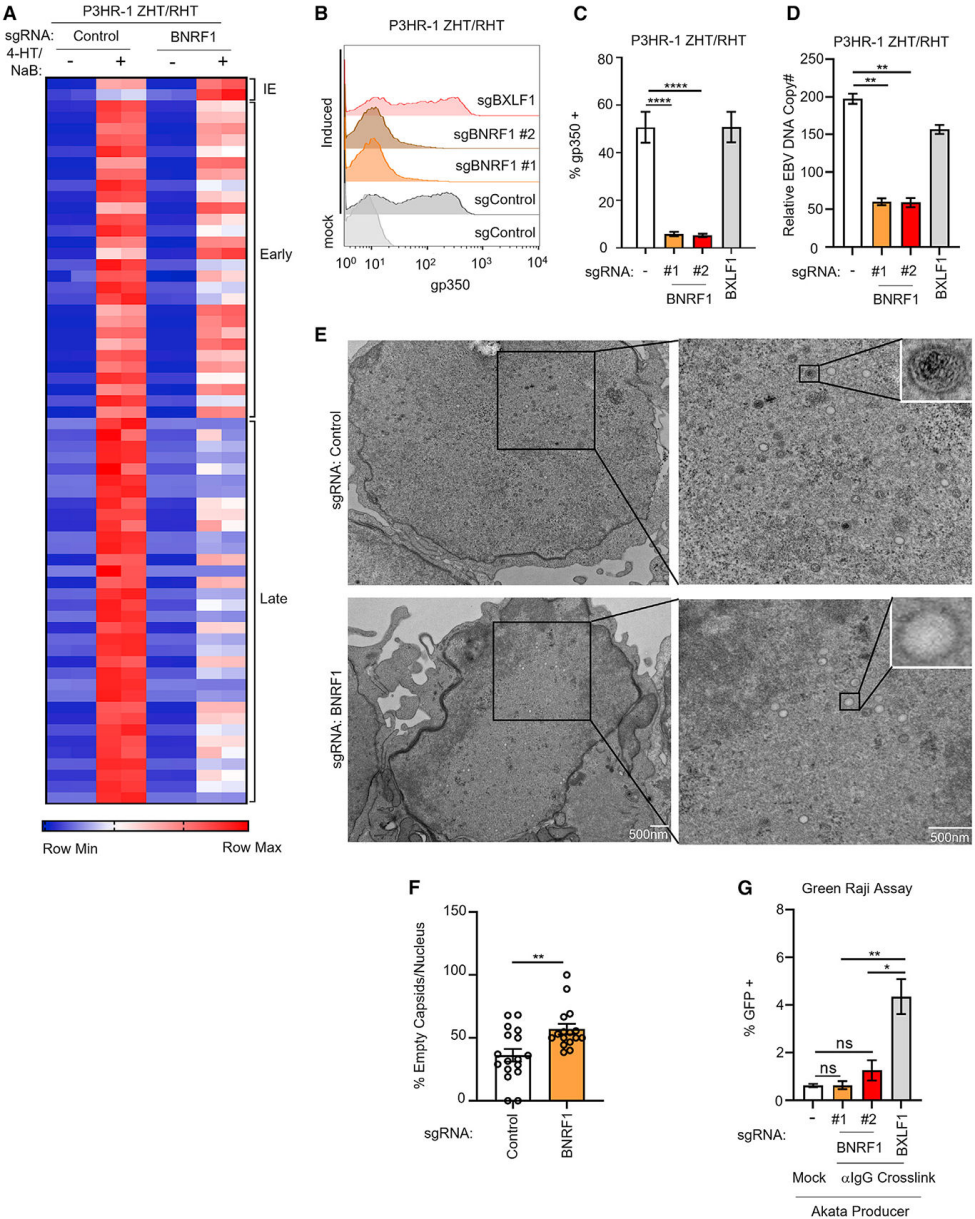
BNRF1 supports late lytic cycle progression, viral DNA replication, and infectious virion production (A) Heatmap of normalized EBV gene expression levels from RNA-seq analysis of P3HR-1 ZHT/RHT cells with control or BNRF1 sgRNA, mock induced or induced into the lytic cycle with 4-HT/NaB for 24 h, from 2 replicates. (B) Flow cytometry analysis of plasma membrane (PM) gp350 expression in P3HR-1 ZHT/RHT cells expressing control BXLF1 or BNRF1 sgRNAs, mock induced or induced for lytic reactivation for 24 h with 4-HT/NaB. (C) PM gp350 in P3HR-1 ZHT/RHT cells obtained as in (B) from 5 replicates. Error bars indicate mean ± SE. (D) qRT-PCR of EBV intracellular genome copy numbers from P3HR-1 cells with BXLF1 or BNRF1 sgRNA induced by 4-HT/NaB for 24 h. Mean ± SE values from 3 replicates are shown. (E) Transmission electron microscopy (TEM) of P3HR-1 ZHT/RHT cells with control or BNRF1 sgRNAs induced into the lytic cycle with 4-HT/NaB for 24 h. Scale bar, 500 nm. (F) Percentage of empty capsids observed from TEM analysis of P3HR-1 ZHT/RHT cells with control versus BNRF1 sgRNA as in (E). Data are from 20 randomly chosen nuclear fields. (G) Mean ± SEM from 3 replicates of green Raji assay analysis of infectious EBV titers from EBV+ Akata cells with BXLF1 or BNRF1 sgRNA induced by immunoglobulin G (IgG) crosslinking for 48 h from 3 replicates. ****p < 0.0001, **p < 0.01. See also Figure S4.

To gain insights into BNRF1 roles in support of lytic replication, we performed RNA sequencing (RNA-seq) on control versus BNRF1 KO P3HR-1 ZHT/RHT cells prior to and 24 h after lytic induction by addition of 4-HT and sodium butyrate (NaB). Although expression of EBV early lytic genes was somewhat lower in *BNRF1*-edited cells, late gene expression was strongly reduced (Figures 4A and S4A), suggesting that BNRF1 may be important for sustaining late gene expression. qPCR analysis of the late lytic genes BVRF1 and BcLF1 validated this result (Figure S4B). To control for CRISPR editing of the EBV episome, which is present at higher copy numbers than host chromosomes, we used an sgRNA targeting the lytic gene *BXLF1*, which is not essential for EBV replication (Meng et al., 2010). Reduction of EBV late gene gp350 expression was similarly evident at the protein level, where BNRF1 sgRNAs significantly reduced gp350 levels relative to those seen in cells with the control BXLF1 sgRNA (Figures 4B and 4C). Robust *BXLF1* editingwas confirmed by T7E1 assay(FiguresS4C). Similar results were observed in EBV+ Akata cells triggered for lytic reactivation by anti-immunoglobulin crosslinking (Figures S4D-S4F).

Because EBV late gene transcription requires continuous lytic DNA replication (Li et al., 2018), we tested whether BNRF1 loss affects EBV genome copy numbers following lytic reactivation. We observed a significant reduction in EBV DNA levels in P3HR-1 ZHT/RHT and EBV+ Akata cells expressing independent BNRF1 sgRNAs relative to levels in cells with non-targeting or BXLF1 control sgRNAs (Figures 4D and S4G). This result was unexpected, given the modest effects on expression of EBV early genes, which encode factors that replicate viral DNA.

To further characterize BNRF1 KO effects on EBV lytic replication, we utilized transmission electron microscopy (TEM). Intriguingly, TEM demonstrated a significant reduction in nuclear capsids filled with electron-dense material characteristic of EBV genomic DNA (Figures 4E and 4F). Approximately 60% of nuclear capsids lacked electron-dense material in *BNRF1*-edited cells compared with ~40% in control cells (Figure 4F). Because capsids are encoded by EBV late genes, we suspect that they were synthesized at the onset of the late phase but that their expression was not sustained at later time points, as measured by RNA-seq. Impaired encapsidation may reflect diminished EBV DNA production, a defect in trafficking of DNA to or insertion into viral capsids as a result of diminished late gene expression, or perhaps SMC5/6-mediated EBV lytic DNA compaction.

We next used the green Raji infection assay (Altmann and Hammerschmidt, 2005) to test whether BNRF1 editing reduced titers of EBV released from lytic cells. This assay leverages the GFP marker expressed by EBV bacterial artificial chromosome (BAC) genomes present in producer cells. Superinfection of Raji cells results in a GFP signal. We found that expression of BNRF1 sgRNA significantly reduced titers of infectious EBV produced by EBV+ Akata cells relative to levels observed in control BXLF1 sgRNA-expressing cells (Figures 4G and S4H). These results suggest that BNRF1 is important for late lytic cycle progression, viral DNA packaging into nuclear icosahedral capsids, and, ultimately, secretion of infectious virions.

### BNRF1 is critical for EBV RC formation

**Figure 5.**
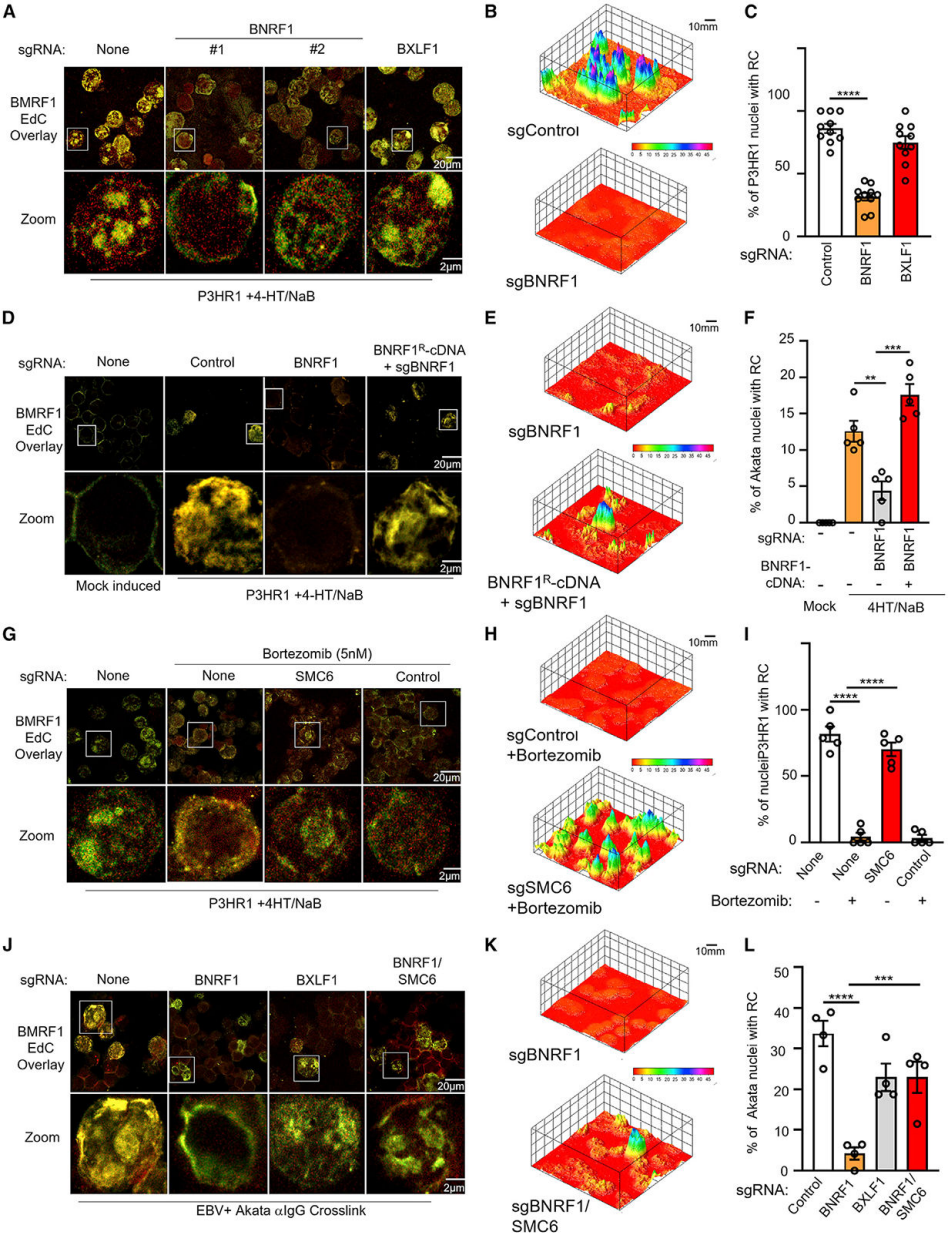
BNRF1 is critical for viral RC formation (A) Immunofluorescence analysis of replication compartments (RCs), as judged by EdC-labeled DNA and staining for BMRF1 in P3HR-1 ZHT/RHT cells that expressed the indicated sgRNAs and that were induced by 4-HT/NaB for 24 h. Magnified images of cells in white boxes are shown at the bottom. (B) 3D reconstruction of EdC and BMRF1 signals as in (A), using the ImageJ Interactive 3D Surface Plot package. The scale bar indicates fluorescence intensity. (C) Mean ± SEM percentages of nuclei with RCs from 3 replicates as in (A), using data from 10 randomly selected panels of 150 nuclei and ImageJ. (D) RC immunofluorescence analysis as in (A), using P3HR-1 ZHT/RHT cells that expressed the indicated sgRNAs and rescue BNRF1 cDNA. (E) 3D reconstruction of EdC and BMRF1 signals as in (D), using the ImageJ Interactive 3D Surface Plot package. (F) Mean ± SEM percentages of nuclei with RCs from 3 replicates as in (D), using data from 5 randomly selected panels of 75 nuclei and ImageJ. (G) RC immunofluorescence analysis as in (A), using P3HR-1 ZHT/RHT cells that expressed no sgRNA, control or SMC6 sgRNA, and were treated with bortezomib, as indicated. (H) 3D reconstruction of EdC and BMRF1 signals as in (G), using the ImageJ Interactive 3D Surface Plot package. (I) Mean ± SEM percentages of nuclei with RCs from 3 replicates as in (G), using data from 5 randomly selected panels of 75 nuclei and ImageJ. (J) RC immunofluorescence analysis as in (A), using P3HR-1 ZHT/RHT cells that expressed the indicated sgRNAs. (K) 3D reconstruction of EdC and BMRF1 signals as in (J), using the ImageJ Interactive 3D Surface Plot package. (L) Mean ± SEM percentages of nuclei with RCs from 3 replicates as in (D), using data from 4 randomly selected panels of 60 nuclei and ImageJ. See also Figures S5-S7.

Ongoing EBV DNA replication is important for maintenance of EBV RCs (Li et al., 2018). Because late gene transcription and DNA replication were unexpectedly reduced in *BNRF1*-edited cells, we hypothesized that the SMC5/6 cohesin complex can recognize and restrict EBV RC, serving a key innate immune role. P3HR-1 ZHT/RHT cells induced for lytic replication were treated with the cytosine homolog 5-ethynyl-2′-deoxycytidine (EdC). A click chemistry approach then allowed EdC biotinylation for streptavidin-based visualization (Qu et al., 2011). Because EBV lytic replication causes growth arrest, most EdC incorporation results from EBV lytic DNA incorporation and serves to highlight RCs. To further demarcate RCs, cells were concurrently immunostained for BMRF1 (Nagaraju et al., 2019). BNRF1 KO by independent sgRNAs, but not control BXLF1 KO, strongly reduced EdC incorporation in P3HR-1 cells induced for lytic replication. Likewise, BNRF1 KO caused striking BMRF1 redistribution from a globular pattern in control BXLF1 KO cells to a perinuclear pattern (Figures 5A and S5A). BNRF1 KO did not change background EdC incorporation (Figure S5B). 3D image reconstruction showed that globular RC structures were diminished in BNRF1 KO cells (Figures 5B, 5C, and S5C). On-target BNRF1 sgRNA effects on RC formation were confirmed by cDNA rescue, using a BNRF1 construct with silent mutation at the protospacer-adjacent motif to abrogate Cas9 cutting (Figures 5D-5F, S5D, and S5E).

We reasoned that proteasome inhibition should phenocopy BNRF1 KO effects on the EBV lytic cycle by stabilizing SMC5/6 complexes. Indeed, treatment with 5 nM bortezomib reduced the number of RCs formed in P3HR-1 ZHT/RHT cells by nearly 80% relative to levels in control cells. Because proteasome inhibitors have pleotropic roles, we next investigated whether SMC6 KO could rescue bortezomib effects on RCs. Intriguingly, CRISPR SMC6 editing nearly completely rescued RC formation in bortezomib-treated cells (Figures 5G-5I, S6A, and S5B), suggesting that degradation of SMC6 is critical for EBV RCs. Likewise, SMC6 KO significantly rescued RC formation in EBV+ Akata BNRF1 KO cells triggered for lytic reactivation by immunoglobulin crosslinking (Figures 5J-5L, S6C, and S6D) and also restored late gene gp350 expression (Figures S6E and S6F). Titers produced by BNRF1/SMC6 double KO were similar to those produced by EBV+ Akata cells expressing paired control sgRNA against host and viral genome sites and stimulated by immunoglobulin crosslinking (Figure S6G).

To test the extent to which BNRF1 subversion of ATRX/DAXX might also be important for lytic replication, we used CRISPR to create BNRF1/ATRX double KO cells. In contrast to BNRF1/SMC6 double KO, BNRF1/ATRX double KO could not rescue plasma membrane gp350 expression, which is dependent on lytic EBV DNA replication in RCs. ATRX KO also failed to rescue production of infectious virions in BNRF1/ATRX double KO EBV+ Akata cells (Figures S6E and S6F). Our data suggest that SMC5/6 degradation, rather than targeting of ATRX/DAXX complexes, is the biologically key BNRF1 target that supports EBV lytic replication. (Figures S6G and S6H).

The SMC5/6 cohesin complex has multiple activities, including Small Ubiquitin-like Modifier (SUMO) ligase, DNA entrapment, and compaction (Yu et al., 2021). We therefore used CRISPR to investigate whether SMC5/6 components in addition to SMC6 were essential for restriction of RCs. KO of the SUMO ligase subunit NSE2 partially restored RC formation in bortezomib-treated cells, suggesting that SUMOylation of a host or viral RC component is involved (Figures S7A-S7C). KO of the SMC5 subunit, which forms long filamentous structures with SMC6 (Figure 1B), rescued RC formation in bortezomib-treated cells to a similar extent as NSE2 KO. Similar results were obtained with KO of the non-SMC element (NSE) subunit NSE3, which forms a subcomplex together with NSE1 and NSE4 that binds to dsDNA (Figures S7A-S7C). These effects were also BNRF1 dependent because KO of SMC5 or NSE2 could at least partially rescue RC formation in BNRF1-edited EBV+ Akata B cells (Figures S7D-S7G). These results suggest that EBV relies on BNRF1 to prevent entrapment as well as a SUMOylation event that counteracts RC.

### SMC5/6 associates with R-loops in EBV RCs

A key question is how the SMC5/6 cohesin complex recognizes viral DNA. *In vitro*, SMC5/6 preferentially binds non-B-form DNA and supercoiled substrates (Gutierrez-Escribano et al., 2020; Serrano et al., 2020). These can include R-loops, which are stable RNA:DNA hybrid triple-stranded structures (Allison and Wang, 2019; Wang et al., 2018). Conditional inactivation of the NSE4 subunit in budding yeast causes increased levels of R-loops (Chang et al., 2019), and R-loops have key roles at gammaherpesvirus *oriLyts* in DNA replication by allowing initial DNA strand separation and core replication protein loading (Rennekamp and Lieberman, 2011). We therefore hypothesized that BNRF1 is necessary to prevent SMC5/6 recognition of EBV lytic genomic R-loops.

**Figure 6.**
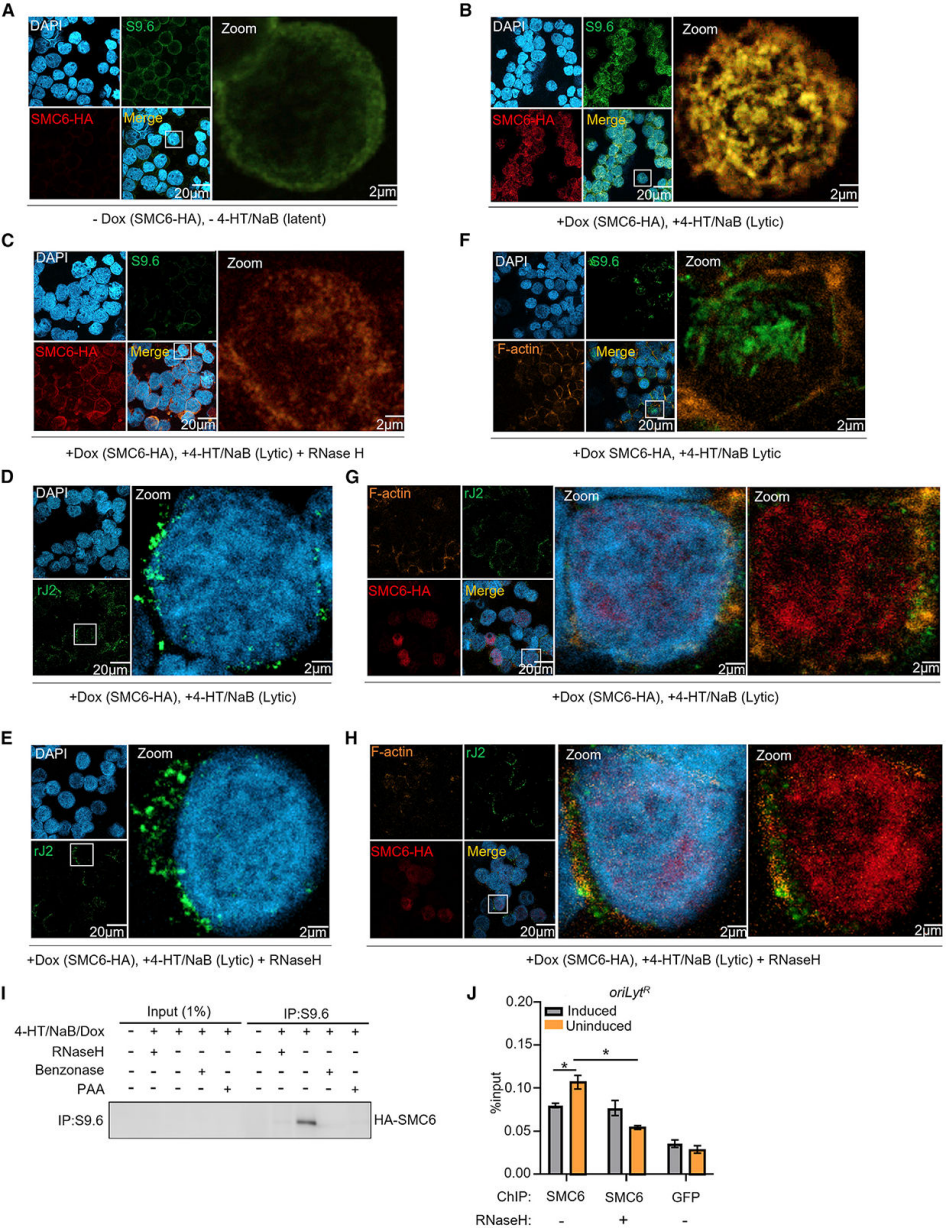
SMC6 associates with EBV genomic R-loop regions in the absence of BNRF1 (A) Confocal immunofluorescence analysis of P3HR-1 ZHT/RHT cells stained with S9.6, anti-SMC6-HA, or DAPI. S9.6 recognizes R-loops and double-stranded RNA (dsRNA). Cells were not induced for exogenous SMC6-HA (−dox) or for lytic reactivation (−4-HT/NaB). Representative of 3 independent experiments. (B) Confocal analysis of P3HR-1 ZHT/RHT cells treated with dox (5 μg/mL) to induce exogenous SMC6 expression and with 4-HT/NaB for 9 h to induce EBV lytic reactivation, as indicated. Cells were then stained with S9.6, anti-HA-SMC6, or DAPI. Representative of 3 independent experiments. (C) Confocal analysis of cells as in (B) that were pre-incubated with RNaseH (5.5 U/μg DNA) prior to staining with S9.6, anti-HA, or DAPI. Representative of 3 independent experiments. (D) Confocal analysis of cells as in (B) that were stained with the anti-dsRNA antibody rJ2 or DAPI. Representative of 3 independent experiments. (E) Confocal analysis of cells as in (B) that were pre-incubated with RNaseH (5.5 U/μg DNA) prior to staining with the anti-dsRNA antibody rJ2 or DAPI. Representative of 3 independent experiments. (F) Confocal analysis of cells as in (B) that were stained with S9.6, anti-F-actin, or DAPI. Representative of 3 independent experiments. (G) Confocal analysis of cells as in (B) that were stained with the anti-dsRNA antibody rJ2, anti-HA-SMC6, anti-F-actin, or DAPI, as indicated. Representative of 3 independent experiments. (H) Confocal analysis of cells as in (B) that were pre-incubated with RNaseH (5.5 U/μg DNA) prior to staining with the anti-dsRNA antibody rJ2, anti-HA-SMC6, anti-F-actin, or DAPI. Representative of 3 independent experiments. (I) Immunoblot analysis of 1% input and S9.6-immunopurified complexes from P3HR-1 ZHT/RHT cells induced for exogenous HA-SMC6 and treated with 4-HT/NaB and phosphonoacetic acid (PAA; 200 μg/mL) for 9 h, as indicated. Samples were treated with RNaseH (5.5 U/μg DNA) or benzonase (5 U/μg DNA) prior to immunoprecipitation (IP), as indicated. (J) Mean ± SEM values from 3 replicates of ChIP-qPCR analysis of SMC6 occupancy at the EBV genomic *oriLyt*^R^ region. Anti-HA ChIP was performed on chromatin from EBV+ Akata cells with dox induced (5 μg/mL) SMC6-HA or GFP-HA expression following 48 h of αIgG crosslinking, followed by qPCR using *oriLyt*^R^ region-specific primers. *p < 0.05.

To gain insights into the molecular mechanism by which the SMC5/6 complex inhibits the EBV lytic cycle, we stained cells with the monoclonal antibody S9.6, which recognizes R-loops and double-stranded RNA (dsRNA). In uninduced P3HR-1 ZHT/RHT cells, SMC6 highly co-localized with the S9.6 signal in a peripheral nuclear distribution, presumably co-localizing with structures of host cell origin. In contrast, lytic induction redistributed the S9.6 signal to nuclear foci. Conditionally overexpressed SMC6, which, we reasoned, was expressed at a high enough level to overcome targeting by BNRF1, also redistributed to co-localizing nuclear puncta in reactivated cells (Figures 6A and 6B).

To more specifically analyze the S9.6 signal specific to R-loops, we treated cells with RNaseH, an endonuclease that digests R-loops. RNaseH strongly diminished the overall S9.6 signal, suggesting that it largely recognized R-loops in cells triggered for lytic replication (Figure 6C). RNaseH also changed the subnuclear distribution of SMC6, suggesting that SMC6 substantially homes to RNA:DNA hybrids in lytic cells in the absence of RNaseH. We next immunostained cells with the anti-dsRNA monoclonal antibody rJ2 in the absence or presence of RNaseH pre-treatment. In contrast with that of S9.6, the rJ2 dsRNA signal was not substantially affected by RNaseH (Figures 6D and 6E). Moreover, SMC6 and S9.6 signals overlapped with nuclear DAPI rather than with cytoplasmic F-actin staining, whereas rJ2 signals were predominantly perinuclear and cytoplasmic (Figures 6F-6H). A large proportion of HA-SMC6 induced by 9 h of doxycycline treatment co-immunoprecipitated with S9.6 (Cristini et al., 2018) in P3HR-1 ZHT/RHT concurrently induced for lytic reactivation (Figure 6I). Whole-cell HA-SMC6 abundance was low because of the short period of doxycycline induction and also because of its destabilization by EBV lytic reactivation, resulting in weak signals in lanes 2–5. Importantly, R-loop destruction by addition of RNaseH prevented SMC6 pull-down by S9.6. As a positive control, addition of benzonase, which degrades all forms of nucleic acid, also perturbed SMC6 co-immunoprecipitation with S9.6. Similarly, blockade of EBV lytic genome synthesis by addition of phosphonoacetic acid (PAA) prevented SMC6 co-immunoprecipitation (Figure 6I).

**Figure 7.**
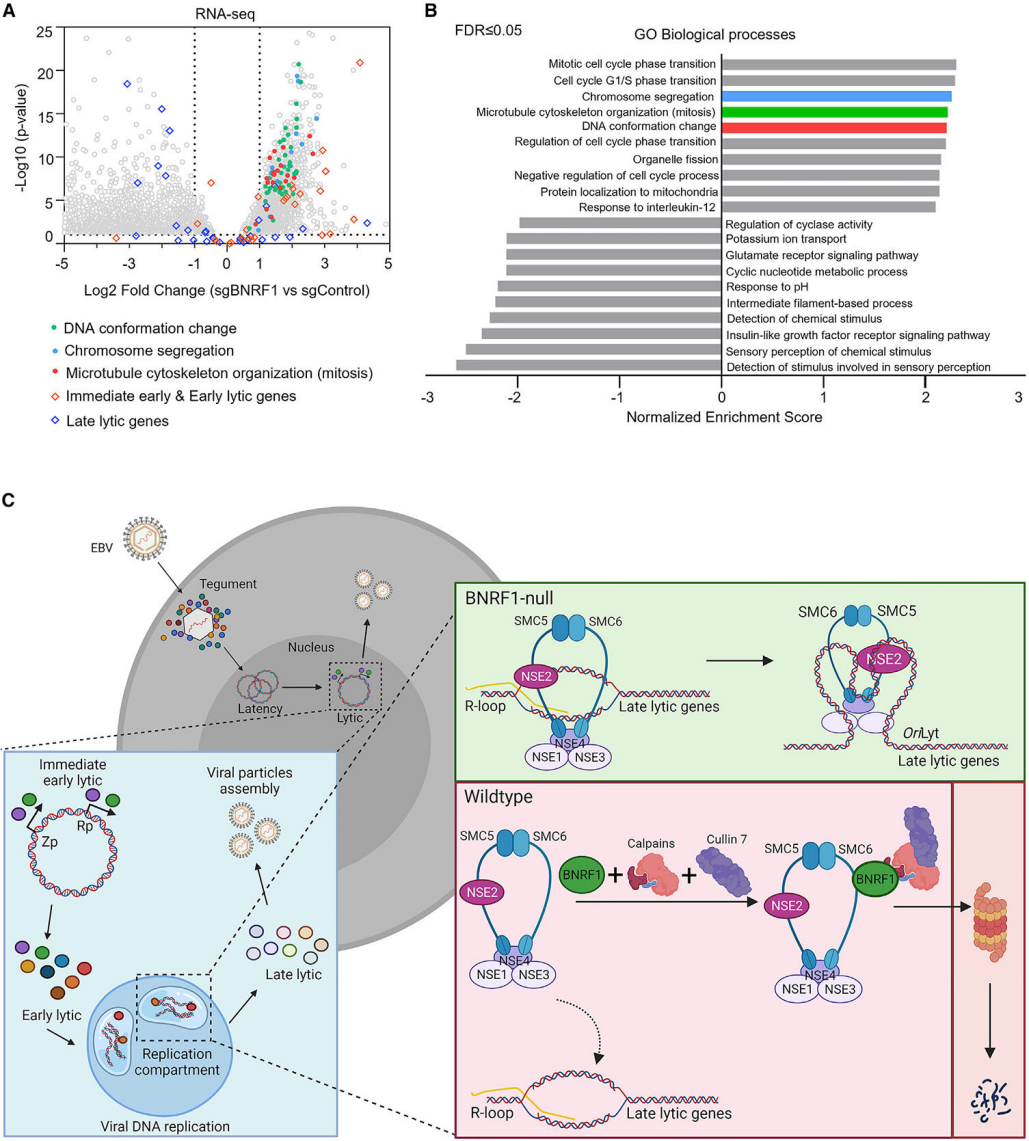
BNRF1 KO effects on lytic cycle host and viral gene expression (A) RNA-seq volcano plot analysis of host mRNA abundance in P3HR-1 ZHT/RHT cells that expressed BNRF1 versus control sgRNAs and that were induced by 4-HT/NaB for 24 h, as in Figure 4A. Circles represent individual host mRNA values. Transcripts encoding proteins involved in DNA conformation change, chromosome segregation, and mitosis are highlighted. (B) Gene Ontology (GO) biological process enrichment analysis of host mRNAs differentially expressed in P3HR-1 ZHT/RHT cells with BNRF1 versus control sgRNAs and induced by 4-HT/NaB for 24 h, as in Figure 4A. (C) Schematic of BNRF1 evasion of SMC5/6 complexes in lytic replication. BNRF1 produced upon late lytic cycle progression associates with calpain and Cul7 to target the SMC5/6 cohesin complex for ubiquitin-proteasome pathway degradation. In the absence of BNRF1, the SMC5/6 complex associates with EBV genomic R-loops, including at the *oriLyt* and perhaps additional non-B-form DNA structures formed in lytic replication to prevent sustained late gene expression, RC formation, EBV lytic genome encapsidation, and infectious virion assembly.

To directly test whether SMC6 associates with EBV lytic genomic R-loops, we performed chromatin immunoprecipitation (ChIP) for HA-epitope tagged SMC6 versus GFP control, followed by qPCR for EBV *oriLyt*^R^, which has been found previously to contain an R-loop structure in reactivated cells (Rennekamp and Lieberman, 2011). ChIP-qPCR identified that SMC6 associated with *oriLyt*^R^ in cells induced for lytic replication, and this association was perturbed by addition of RNaseH (Figure 6J). Interestingly, DNA conformation change, chromosome segregation, and telomere organization were among the most enriched pathways identified by Gene Ontology analysis of differentially expressed host genes in control versus BNRF1 KO P3HR-1 ZHT/RHT cells induced for lytic replication (Figures 7A and 7B). Given the well-defined SMC6 roles in chromosome segregation, genome stability, alternative lengthening of the telomere, and the ability of telomeres to form R-loop structures (Graf et al., 2017), we speculate that these mRNA changes directly arise from compensatory responses to the stabilization of SMC6 in the absence of BNRF1. These results support a model where, in the absence of BNRF1, SMC6 complexes recognize and occupy R-loops formed in the late lytic EBV cycle (Figure 7C).

## DISCUSSION

To periodically reactivate in immunocompetent hosts, EBV circumvents multiple layers of host defense, including intrinsic immune responses that sense and respond to foreign viral DNA. How EBV RCs evade nuclear intrinsic immune pathways has remained an important question. In contrast to lipid-enclosed viral RCs used by positive-sense RNA viruses that shield viral nucleic acids, EBV and other herpesviruses utilize nuclear membrane-less RCs that are accessible to intrinsic immune responses. Our results position BNRF1 and the SMC5/6 cohesin complex as central players in the EBV host/pathogen interface (Figure 7C) and suggest that all herpesviruses may need to counteract SMC5/6 to support RC formation and lytic virus replication.

RCs are seeded by single, ~170-kb EBV genomes that synthesize thousands of EBV genome copies. Our results suggest that BNRF1 circumvents SMC5/6 cohesin complexes from recognizing and suppressing lytic EBV genomes that seed and/or drive RC expansion. A similar phenomenon has also been reported in adenovirus-infected cells, in which the viral E4 early protein targets SMC6 for degradation. In the absence of E4, SMC6 associates with replicating adenoviral dsDNA genomes and impairs viral lytic DNA replication (Dybas et al., 2021). At early time points of lytic reactivation, EBV DNA templates are limiting for genome amplification and RC expansion (Nagaraju et al., 2019), and pharmacological blockade of lytic DNA replication collapses RCs (Li et al., 2018). Thus, in the absence of BNRF1, SMC5/6 may suppress RC expansion by blocking lytic genome synthesis. This effect may also contribute to the increased numbers of empty capsids observed in BNRF1 KO cells, together with perturbed late gene expression. We speculate that BNRF1 has similarly important roles in supporting lytic replication in epithelial cells as well as in the establishment of B cell latency, given profound SMC6 depletion in each of these settings. Indeed, in the absence of BNRF1, EBV latency gene expression is highly attenuated in newly infected B cells (Feederle et al., 2006; Tsai et al., 2011), which may result not only from repressive effects of histone 3.3 by α-Thalassemia/mental Retardation syndrome X-linked/Death Domain Associated Protein (ATRX/DAXX) but also from SMC5/6 loading.

Use of a tegument protein, which is packaged in the virion and therefore primed to disarm the SMC5/6 complex without need for *de novo* EBV transcription or translation, provides EBV with a stealth approach. Likewise, BNRF1 expression during lytic replication depletes SMC5/6 at a time when it would otherwise recognize and counteract lytic genomes. This strategy provides a defined window during which EBV disrupts the SMC5/6 complex, typically in growth-arrested newly infected or lytic cells, perhaps limiting deleterious effects to host cells. However, EBV lytic replication is increasingly linked to cancer (Munz, 2019; Shannon-Lowe and Rickinson, 2019). For instance, elevated antibody titers against EBV lytic antigens are predictive of nasopharyngeal carcinoma (Chien et al., 2001). Similarly, the M81 EBV strain isolated from an individual with nasopharyngeal carcinoma exhibits elevated levels of lytic replication (Tsai et al., 2013). Lytic reactivation contributes to transformed B cell outgrowth *in vivo* in humanized mouse models (Hong et al., 2005). Because SMC5/6 has key roles in host chromosome biology and DNA damage response, our results provide a mechanism for the observation that BNRF1 can induce centrosome amplification and chromosomal instability in newly infected B cells (Shumilov et al., 2017).

BNRF1 subversion of SMC5/6 may provide a mechanism by which EBV is associated with cancers, including by “hit-and-run” abortive infection. Such BNRF1 effects may be connected to the clinical observation that infectious mononucleosis increases the risk of EBV+ Hodgkin’s lymphoma and EBV-non-Hodgkin’s lymphoma over the first year after infection (Ekstrom-Smedby, 2006; Hjalgrim et al., 2003). Atypical chromosomal structures and nuclear morphology are often noted in tissue of individuals with active EBV infection, including acute infectious mononucleosis (Watt et al., 1977). Although BNRF1 also disrupts ATRX/DAXX complexes (Tsai et al., 2011), this observation does not explain the chromosomal instability observed during EBV infection and lytic replication because DAXX only transiently colocalizes with centromeres (Morozov et al., 2012). In contrast, BNRF1 overexpression results in centrosome overduplication (Shumilov et al., 2017).

In the absence of viral evasion, the SMC5/6 complex can restrict expression of dsDNA viral genomes, including those of herpes simplex virus, human papillomavirus, HBV, and unintegrated HIV cDNA (Decorsiere et al., 2016; Dupont et al., 2021; Gibson and Androphy, 2020; Murphy et al., 2016; Xu et al., 2018). How SMC5/6 recognizes viral DNA has remained a key question because specific sequences or structures that recruit SMC5/6 to viral genomes have not been identified. Recent *in vitro* studies suggest that yeast and human SMC5/6 cohesin complexes preferentially bind to non-B-form DNA, including highly supercoiled, catenated, and plectoneme structures (Gutierrez-Escribano et al., 2020; Serrano et al., 2020). We present data showing that, in cells undergoing lytic replication in the absence of BNRF1, SMC5/6 highly associated with the signal from the monoclonal antibody S9.6, which recognizes R-loops and dsRNA.

R-loops are triple-stranded RNA:DNA hybrid structures that have been linked to genome instability (Bayona-Feliu et al., 2021; Chen et al., 2017), whereas SMC5/6 have major roles as guardians of chromosome stability. Our experiments with RNaseH pretreatment support R-loops, rather than dsRNA, as the major SMC5/6 cohesin complex target in lytic B cells. EBV origins of lytic replication contain G-rich regions, which are prerequisites for RNA:DNA hybrid structures, and R-loops form at these regions in lytic EBV genomes (Rennekamp and Lieberman, 2011). Computational analysis performed on 6,000 viruses suggests that more than 70% of dsDNA viruses encode R-loop-forming sequences, including all viruses in the Herpesvirales order (Wongsurawat et al., 2020). These observations suggest a potentially central mechanism of SMC5/6 recruitment to herpesvirus genomes in the absence of viral evasion. In contrast, SMC5/6 is recruited to unintegrated HIV proviral genomes by the adaptor protein SLF2, perhaps because such cDNA does not have non-B-form DNA regions such as R-loops (Dupont et al., 2021).

How SMC5/6 disrupts expression of EBV late genes remains an objective for future studies. Our data support a model where, upon recognition of EBV R-loop and perhaps other non-B-form DNA structures formed in lytic replication, SMC5/6 complexes are loaded onto circular EBV genome templates for lytic replication in the absence of BNRF1. This may impair production of linear lytic genomes, resulting in diminished late gene expression and decreased viral loads. It remains plausible that SMC5/6 are also loaded onto linear genomes produced by the EBV DNA polymerase to impair their packaging into viral capsids, perhaps by DNA compaction.

Unexpectedly, BNRF1 juxtaposes calpain and SMC5/6 complexes. Calpains are Ca^2+^-dependent cysteine proteases that can mark substrates for proteasomal degradation by the Arg/N-end rule pathway (Piatkov et al., 2014; Shemorry et al., 2013; Varshavsky, 2011). We suspect that calpain SMC6 cleavage exposes an N-terminal degron signal that leads to its Cul7-mediated ubiquitin proteasome degradation. It is noteworthy that BNRF1 targets SMC5/6 and ATRX/DAXX by distinct mechanisms. Interestingly, the murine gammaherpesvirus homolog tegument protein ORF75c induces PML degradation to support viral gene expression (Ling et al., 2008). It is plausible that different BNRF1 pools carry out these two important antiviral functions. Alternatively, use of distinct BNRF1 surfaces for SMC6 versus DAXX association may allow preferential calpain recruitment to the SMC5/6 interface.

BAC EBV genomic BNRF1 KO did not apparently reduce EBV viral load in a HEK293 cell system (Feederle et al., 2006). HEK293 cells lack multiple DNA sensors, such as Cyclic GMP-AMP Synthase/Stimulator Of Interferon Genes (cGAS/STING), and we observed low levels of SMC6 by immunoblot of HEK293 whole-cell lysates, perhaps obviating the need for BNRF1 in this system. We observed SMC6 loss in AGS gastric carcinoma cells induced for lytic replication (Figure 1D) and suspect that BNRF1 has similarly important roles in support of epithelial cell lytic replication.

How can the late gene BNRF1 be important for expression of other EBV late genes and have a role in DNA replication, which is initiated in the early lytic period? First, although cap analysis of gene expression sequencing (CAGE-seq) studies suggest that BNRF1 is expressed with late gene kinetics in HEK293 cells with EBV BAC genomes (Djavadian et al., 2018), independent studies have also identified its expression in lymphoblastoid cell lines with the latency III program (Abbott et al., 2013; Adhikary et al., 2020). Low basal BNRF1 levels may have important roles even within early phases of B cell lytic cycles. We suspect that an initial burst of late gene synthesis likely produces the empty capsids observed by electron microscopy. Second, although late gene transcription precedes significant increases in EBV DNA copy numbers, ongoing DNA replication is necessary for sustained late gene expression (Li et al., 2018). Therefore, BNRF1 may be required to maintain ongoing late gene expression.

The EBV major tegument protein BNRF1 is critical for defense of membrane-less nuclear RCs against the activity of SMC5/6 cohesins. There is increasing interest in lytic induction approaches that leverage the presence of EBV genomes to sensitize tumors to antiviral agents or to targeted CD8+ T cell killing (Feng et al., 2004). However, induction of virion production carries the risk of delivering BNRF1 to neighboring cells. Therefore, where the goal is to sensitize tumors to adoptively transferred anti-EBV T cells against the highly immunogenic EBV immediate-early BZLF1, it may be prudent to consider use of acyclovir or ganciclovir to block EBV late gene expression. This approach could minimize DNA damage to neighboring cells caused by delivery of BNRF1 and SMC5/6 depletion.

### Limitations of the study

A key limitation of this study is that, to our knowledge, monoclonal S9.6 is the only antibody that visualizes R-loops, but it also recognizes additional structures, in particular dsRNA. We attempted to establish R-loop specificity of our S9.6 results by inclusion of experiments with RNaseH, an endonuclease that cleaves RNA at RNA:DNA hybrids and is therefore specific for R-loop structures. Nonetheless, additional studies should be performed with R-loop-specific reagents as they become available. Similarly, knowledge remains incomplete about early events that enable rapid EBV lytic DNA amplification, perhaps prior to rolling circle amplification. It remains plausible that additional non-B-form DNA structures formed by EBV lytic replication are sensed by SMC5/6, which could include plectoneme structures. Second, the precise mechanism by which SMC5/6 restricts EBV RC and late gene expression in the absence of BNRF1 remain to be established. Additional studies are needed to ascertain whether SMC5/6 topological DNA entrapment is sufficient or whether EBV DNA is compacted by SMC5/6 complexes. Similarly, precise SUMOylation roles, which our CRISPR KO studies suggest to also be important for SMC5/6 antiviral activity, remain to be established. Third, additional studies are required to establish whether BNRF1 targeting of SMC5/6 is necessary for establishment of latency, licensing of EBV oncoprotein induction, and/or the initial phases of growth transformation in newly infected primary human B cells. The recent development of primary human B-cell CRISPR editing techniques should facilitate these studies.

## STAR★METHODS

### RESOURCE AVAILABILITY

#### Lead contact

Further information and requests for resources and reagents should be directed to and will be fulfilled by the Lead Contact, Benjamin E. Gewurz (bgewurz@bwh.harvard.edu).

#### Materials availability

All reagents will be made available on request after completion of a Materials Transfer Agreement.

#### Data and code availability

- All RNA-seq datasets have been deposited to the NIH GEO omnibus (GEO: GSE182349) and are publicly available as of the date of publication. All raw data have been deposited at Mendeley and are publicly available as of the date of publication (Mendeley Data: https://doi.org/10.17632/5v545cw8t7.1). Microcopy data reported in this paper will be shared by the lead contact upon request. Figures were drawn with commercially available GraphPad, Biorender and Microsoft Powerpoint.
- This paper does not report original code.
- Any additional information required to reanalyze the data reported in this paper is available from the lead contact upon request.

### EXPERIMENTAL MODEL AND SUBJECT DETAILS

HEK293T were cultured in DMEM supplemented with 10% FBS and 1% Pen/Strep. AGSiZ were cultured in F-12-Glutomax supplemented with 10% FBS, 1% Pen/Strep, 0.5 μg/mL puromycin and 0.5 mg/mL G418. P3HR-1-ZHT/RHT-Cas9+, EBV+ Akata-Cas9+, BJAB-Cas9+ and Daudi-Cas9+ cells were cultured in RPMI-1640 supplemented with 10% v/v FBS and 1% Pen/Strep. Cas9+ cells were maintained in 5 μg/mL blasticidin. P3HR-1-Z/R-HT were also maintained with 25 μg/mL G418 and 25 μg/mL hygromycin. All cells were incubated at 37°C with 5% CO_2_ and were routinely confirmed to be mycoplasma-negative. Cell lines were authenticated by STIR profiling. cDNA used in this study were cloned into the pLIX-402 or pLX-TRC313 vector. pLIX-402 uses a Tet ONTRE promoter to drive expression of the gene of interest with a C-terminal HA Tag. pLX-TRC313 uses a EF1α promoter to drive expression of the gene of interest with a C-terminal V5 tag. Stable cell lines were generated by lentiviral transduction and antibiotic selection with puromycin (pLIX-402) or hygromycin (pLX-TRC313). Cell lines were then maintained with 0.5 μg/mL puromycin or 25 μg/mL hygromycin.

### METHOD DETAILS

#### Molecular cloning

Unless otherwise specified, all cloning experiments were performed by Gateway recombination. Briefly, 150 ng of the destination vector and donor vector containing the gene of interest were co-incubated with 1X LR Clonase Enzyme Mix (Invitrogen #11789-020) overnight at room temperature. The reaction mixture was then transformed into 50 μl of Stbl3 bacteria, spread on LB plates with ampicillin.

#### CRISPR analysis

CRISPR/Cas9 editing was performed as described (Guo et al., 2020). Briefly, sgRNAs were cloned into pLentiGuide-puro (Addgene plasmid #52963 (Sanjana et al., 2014)) or pLenti-spBsmBI-sgRNA-Hygro (Addgene plasmid #62205 (Pham et al., 2016)) by restriction-ligation, and sequenced verified. Lentiviral transduction in 293T cells were performed as described previously (Ersing et al., 2017). In brief, 293T cells were co-transfected with 500 ng lentiviral plasmid, 400 ng psPAX2 and 150 ng VSV-G plasmids for packaging. Lentivirus produced were filtered with 0.45 μm filter and transduced into P3HR-1-Z/R-HT-Cas9+ and Akata-EBV-Cas9+ cells. Transduced cells were selected for 1 week with puromycin or 2 weeks with hygromycin. CRISPR KOs were verified by western blot analysis or T7EI endonuclease assay (only when antibody is unavailable). sgRNA against the above-mentioned genes are listed in the Table S1.

#### cDNA rescue

BNRF1 rescue cDNA in entry vector, with silent PAM mutations at BNRF1 sg#1, is described in the Table S1. BNRF1 sg#1 targeting sequence are in bold; while PAM sequence and mutations sites are underlined and indicated in red, respectively. Mutations were performed with Q5 Site-Directed Mutagenesis Kit (NEB) and sequence verified. Rescue BNRF1 cDNA in entry vector was cloned into pLX-TRC313 vector (a gift from John Doench) by Gateway recombination. BNRF1 rescue cDNA was confirmed by western blot analysis using anti-V5 tag antibody.

#### BNRF1 deletion mutants

A collection of 300aa BNRF1 cDNA deletion in entry vector were generated with PrimeSTAR GXL Premix (Takara Bio) according to manufacturer’s instructions, using primers listed in the Table S1. Deletions were sequence verified and were cloned into pLX402 vector by Gateway recombination. Expression of BNRF1-mutant were induced by 5 μg/mL doxycycline and verified by western blot analysis using anti-HA antibody.

#### Primary human B-cell isolation and infection

Platelet-depleted venous blood was obtained from the Brigham and Women’s Hospital Blood Bank, following our Institutional Review Board-approved protocol for discarded and de-identified samples. RosetteSep and EasySep negative isolation kits (STEMCELL Technologies) were used sequentially to isolate CD19+ B-cells with modifications made to the manufacturer’s protocols as described previously (Wang et al., 2019b). For proteomic analysis, primary B-cells were infected by B95.8 EBV, as described (Wang et al., 2019a). For validation studies of SMC6 depletion upon B-cell infection, EBV B95.8 virus was produced from 293 cells with the 2089-EBV-BAC by co-transfecting plasmids expressing BZLF1 and BALF4 for 24 h, followed by treatment with 12-O-tetradecanoylphorbol-13-acetate (TPA, 20 ng/mL) and NaB (2 mM) for an addition of 72 h. Supernatant were collected and filtered through a 0.45 μm filter. Virus were concentrated 200-fold by ultracentrifugation. EBV titer was determined experimentally by transformation assay. EBV was added to 1 M purified B cells at an MOI of 0.8 for 72 h, incubated at 37°C with 5% CO_2_. Cells were harvested and 10% were subjected to flow cytometry analysis for infection efficiency, while the remaining were used for western blot analysis.

#### Immunoblot analysis

Whole cell lysates were separated by SDS-PAGE electrophoresis, transferred onto nitrocellulose membrane, blocked with 5% milk in TBST buffer for 1 h and incubated with the corresponding primary antibodies at 4°C overnight. Blots were washed 3 times in TBST solution and were incubated with secondary antibodies for 1 h at room temperature. Blots were then washed 3 times in TBST solution and were developed by incubating with ECL chemiluminescence. Images were captured by Licor Fc platform. All antibodies used in this study are listed in the key resources table.

#### Flow cytometry analysis

Cells were washed once with cold PBS supplemented with 2% v/v fetal bovine serum (FBS). Cells were then incubated with PE-conjugated anti-CD23 (1:250) or Cy5-conjugaged anti-gp350 antibody (1:1000) in 2% FBS v/v, PBS for 30 mins at 4°C. Cell were pelleted, washed twice, resuspended in 2% FBS v/v, PBS into flow cytometry-compatible tubes and processed immediately. Flow cytometric data was acquired with a BD FACSCalibur instrument and analysis was performed with FlowJo V10.

#### Immunofluorescence analysis

Cells dried on glass slides were fixed with 4% paraformaldehyde/PBS solution for 10 mins, permeabilized with 0.5% Triton X-100/PBS for 5 mins and blocked with 1% BSA/PBS for 1 h at room temperature. For experiments involving RNaseH treatment, cells were blocked with 1% BSA/PBS supplemented with 5.5 U/μg DNA RNaseH (NEB) for 1 h at 37°C. Subsequently, cells were incubated with a cocktail of primary antibodies against BMRF1 (0.4 μg/mL), HA (1:1000) or V5 (1:1000) in blocking solution for 1 h at 37°C. Cells were then washed twice with PBS and incubated with a cocktail of secondary antibodies at 1:500 in PBS for 1 h at 37°C in the dark. Finally, cells were washed twice with PBS and were stained/mounted overnight with ProLong™ Gold Antifade Mountant with DAPI. Image and analysis was performed with Zeiss LSM 800 instrument and with Zeiss Zen Lite (Blue) software, respectively. 3D reconstruction of Figure 4A using Arivis Vision4D from ZEISS ZEN lite (blue edition). Image J was used to score the % of nuclei with RC in P3HR-1 cells, using the ImageJ “Particle Analysis” plugin. For 5-Ethynyl-2’-deoxycytidine (EdC) labelling of newly synthesized DNA, EDC (5 μM) was added together with lytic induction stimuli. Click chemistry was performed to conjugate biotin to the EdC, as described previously (Cabral et al., 2018). In brief, subsequent to secondary antibodies staining, cells were washed twice with PBS. Biotin conjugation to EdC was performed by incubating the cells with PBS supplemented with 200 μM CuSo4, 1 mM sodium ascorbate and 25 μg/mL biotin azide for 2 h at 37°C in dark. Cells were then washed twice with PBS and incubated with streptavidin-conjugated antibodies (1:1000) in PBS for 30 mins at 37°C in dark. Finally, cells were washed twice with PBS and were stained/mounted overnight with ProLong™ Gold Antifade Mountant with DAPI.

#### Green Raji and Daudi assay

Green Raji and Daudi assays were performed as previously described (Altmann and Hammerschmidt, 2005; Pich et al., 2019). In brief, EBV lytic replication was induced by anti-human IgG (15 μg/mL) and supernatant were collected and filtered through 0.8 μm filters at 72 h post induction. 0.1 million/mL Raji or Daudi cells were infected at MOI 0.9. At 24 h post infection, culture media were exchanged to fresh RPMI supplemented with 10% FBS and cells were treated with 20 ng/mL tetradecanoyl phorbol acetate (TPA) and 3 mM NaB for another 48 h. Cells were collected and the percentage of GFP+ cells were determined by flow cytometry.

#### Quantification of EBV copy number

Intracellular EBV genome copy # were quantified by qPCR analysis. For intracellular viral DNA extraction, total DNA from 1x10^6^ cells were extracted by the Blood & Cell culture DNA mini kit (Qiagen). Extracted DNA were diluted to 10 ng/μl and were subjected to qPCR targeting the *BALF5* gene. Serial dilutions of pHAGE-BALF5 plasmid at 25 ng/μl were used to generate the standard curve. Viral DNA copy number was calculated by substituting sample Cq values into the regression equation dictated by the standard curve. qPCR primer sequences used for DNA copy number quantification are listed in the Table S1.

#### T7 endonuclease I (T7EI) assay

T7EI assay was performed using EnGen Mutation Detection Kit (NEB) following manufacturer’s protocol. Briefly, genomic DNA was first extracted from cells expressing sgControl or sgRNA that targets *BXLF1*. Cas9-targeted regions were then PCR amplified. The amplified products were separated by gel electrophoresis, extracted, and purified using QIAquick® Gel Extraction Kit (Qiagen). Equal amount of PCR products from control and BXLF1 KO samples were mixed and were added with 1 μl NEB2 buffer. The mixture was then subjected to 95°C for 10 mins and then cooled down to 4°C at a cooling rate of 0.1 °C/ s in a Thermocyler. 0.25U T7EI was added to the product and was incubated for 1 h at 37°C. The final reaction product was then separated by gel electrophoresis. Images were captured using Licor Fc platform.

#### Co-immunoprecipitation analysis

Expression of SMC6, BNRF1 or the collection of BNRF1 mutants were induced by the addition of 5 μg/mL doxycycline. Bortezomib was added to prevent the degradation of SMC6 during BNRF1 expression or EBV lytic induction. 150 M cells were harvested and was lysed in cold lysis buffer (1% v/v NP40, 150 mM Tris, 300 mM NaCl in dH2O) supplemented with 1X complete™ EDTA-free protease inhibitor cocktail (Sigma), 1 mM Na3VO4 and 1 mM NaF for 1 h at 4°C with rotation. Lysed cells were pelleted, and lysates were incubated with anti-HA tag magnetic beads (Pierce, Thermo) at 4°C overnight. Beads were washed with lysis buffer for four times and were eluted using 1X SDS loading buffer incubated for 10 mins at 95°C. Proteins were separated by SDS-PAGE gel and transferred to nitrocellulose membranes. Subsequent procedures were similar to that mentioned in “Western blot analysis”

#### Poly-ubiquitinylation co-immunoprecipitation analysis

Cells were induced into lytic replication by 2 mM 4-HT/500 μM NaB and were either untreated or treated with 5 nM Bortezomib or 50 μM Calpeptin for 16 h. 150 M cells were harvested and was lysed in cold lysis buffer (1% v/v NP40, 150 mM Tris, 300 mM NaCl in dH2O) supplemented with 1X cOmplete™ EDTA-free protease inhibitor cocktail (Sigma), 1 mM Na3VO4, 1 mM NaF, 1 mM PMSF, 4 mM 1, 10 o-phenanthroline, 2 mM sodium pyrophosphate and 1 mM EDTA for 1 h at 4°C with rotation. Lysed cells were pelleted and precleared with protein A/G magnetic beads (Pierce, Thermo). Precleared lysate was then incubated with antipoly-ubiquitin antibody (Cell signaling, P4D1) for 1 h at 4°C with rotation. Protein A/G magnetic beads were then added to the immunocomplex and were incubated at 4°C overnight. Beads were washed with lysis buffer for four times and were eluted using 1X SDS loading buffer incubated for 10 mins at 95°C. Proteins were separated by SDS-PAGE gel and transferred to nitrocellulose membranes. Subsequent procedures were similar to that mentioned in “Western blot analysis”.

#### R-loop co-immunoprecipitation analysis

SMC6 expression was induced by the addition of 5 μg/mL doxycycline to P3HR-1-Z/R-HT-Cas9+ cells (established with pLX402-SMC6). EBV lytic cycle were induced with procedures mentioned in “reactivation of EBV lytic cycle”. 200 μg/mL PAA was added where indicated. R-loop-IP was performed as described previously (Cristini et al., 2018). Briefly, 50 M of these cells were harvested. They were non-crosslinked and were lysed in lysis buffer (85 mM KCl, 5 mM PIPES (pH 8.0), and 0.5% v/v NP-40) for 10 mins at 4°C with rotation. Pelleted nuclei were resuspended in resuspension buffer (10 mM Tris-HCl pH 7.5, 200 mM NaCl, 2.5 mM MgCl_2_, 0.2% w/v sodium deoxycholate [NaDOC], 0.1% v/v SDS, 0.05% w/v sodium lauroyl sarcosinate [Na sarkosyl] and 0.5% v/v Triton X-100). The extracts were then sonicated (10 s on, 10 s off, 10 cycles) by an ultra-sonication processor (Diagenode, USA). The sonicated extracts were then diluted 1:2 in RSB+T buffer (10 mM Tris-HCl pH 7.5, 200 mM NaCl, 2.5 mM MgCl_2_ and 0.5% v/v Triton X-100) and were subjected to IP with the S9.6 antibody (Millipore) together with protein A/G magnetic beads (Pierce, Thermo) that were washed three times with RSB+T buffer and pre-blocked with 0.5% BSA-PBS for 2 h at 4°C. Where indicated, 5.5U RNaseH per μg of DNA or 1 U/μl benzonase were added to the samples before IP for 1 h at 37°C with rotation. Beads were washed four times with RSB+T buffer, twice with RSB buffer and were eluted in 1X SDS loading buffer for 10 mins at 95°C. Eluted proteins were separated by SDS-PAGE with procedures similar to “Western blot analysis”.

#### Chromatin immunoprecipitation (ChIP)-qPCR

Fifty million P3HR-1-Z/-R-HT-Cas9+ (established with either pLX402-SMC6 or pLX402-GFP) were fixed with 1% PFA (at final concentration) for 10 mins at room temperature. 0.4 M (working concentration) of glycine was then added to the fixed cells and incubated for an addition of 5 mins at room temperature. Cells were lysed with lysis buffer (50 mM HEPES-KOH, 150 mM NaCl, 1 mM EDTA, 1% v/v Triton X-100, 0.1% w/v sodium deoxycholate, and 1% v/v SDS) supplemented with cOmplete™ EDTA-free protease inhibitor cocktail (Sigma) for 1 h at 4°C. They were then fragmented by an ultra-sonication processor (Diagenode, USA). Soluble chromatin was diluted and incubated with 40 μl anti-HA magnetic beads (Pierce, Thermo) overnight at 4°C. Beads were extensively washed and eluted with elution buffer (100 mM NaHCO3 and 1% SDS in dH2O). Reverse cross-linking was performed by protease K treatment (40 U/mL) at 65°C overnight. DNA was then purified by QIAquick PCR purification kit (Qiagen). ChIP assay DNA was qPCR quantified and normalized to the percent of input DNA. Primer sequences used for ChIP-qPCR performed in this study are listed in the Table S1.

#### RNA sequencing (RNA-seq) experiments

Total RNA was isolated using RNeasy mini kit (Qiagen) following manufacturer’s instructions. In-column DNA digestion was included to remove residual genomics DNA contamination. To construct indexed libraries, 1 μg of total RNA was used for polyA mRNA-selection using NEBNext Poly(A) mRNA Magnetic Isolation Module (NEB), followed by library construction using NEBNext Ultra RNA Library Prep Kit for Illumina (NEB). Each experimental treatment was performed in biological triplicate. Libraries were multi-indexed, pooled and sequenced on an Illumina NextSeq 500 sequencer using single-end 75 bp reads (Illumina). All raw sequencing reads were first evaluated using FastQC (http://www.bioinformatics.babraham.ac.uk) and confirmed with no significant quality issues. For RNA-seq data analysis, paired-end reads were mapped to human (GENCODE v28) and EBV (Akata) transcriptome and quantified using Salmon v0.8.2 (Patro et al., 2017) under quasi-mapping and GC bias correction mode. Read count table of human and EBV genes was then normalized across compared cell lines/conditions and differentially expressed genes were evaluated using DESeq2 v1.18.1 (Love et al., 2014) under default settings. Pathway analysis was performed by using WebGestalt (WEB-based Gene SeT AnaLysis Toolkit) functional enrichment analysis web tool under default setting (Liao et al., 2019).

#### Transmission electron microscopy

A pellet of cells was fixed for at least 2 h at RT in fixative (2.5% Glutaraldehyde 1.25% Paraformaldehyde and 0.03% picric acid in 0.1 M sodium cacodylate buffer (pH 7.4)), washed in 0.1 M cacodylate buffer and post-fixed with 1% Osmiumtetroxide (OsO4)/1.5% Potassiumferrocyanide (KFeCN6) for 1 h, washed 2x in water, 1x Maleate buffer (MB) 1x and incubated in 1% uranyl acetate in MB for 1 h followed by 2 washes in water and subsequent dehydration in grades of alcohol (10 mins each; 50%, 70%, 90%, 2X10 mins 100%). The samples were then put in propyleneoxide for 1 h and infiltrated overnight in a 1:1 mixture of propyleneoxide and TAAB (TAAB Laboratories Equipment Ltd, https://taab.co.uk). The following day the samples were embedded in TAAB Epon and polymerized at 60°C for 48 h. Ultrathin sections (about 60 nm) were cut on a Reichert Ultracut-S microtome, picked up on to copper grids stained with lead citrate and examined in a JEOL 1200EX Transmission electron microscope or a TecnaiG^2^ Spirit BioTWIN and images were recorded with an AMT 2k CCD camera.

#### Sample preparation for LC/MS analysis

Whole cell lysates were prepared from 400 million P3HR-1 ZHT/RHT cells (per replicate) that were induced into the lytic cycle by 4-HT (400 nM)/NaB (500 μM) for 24 h and that were also conditionally induced to express HA-tagged BPLF1, BOLF1, BLRF2 or BNRF1 by doxycycline (5 μg/mL) for 15 h. Samples were prepared as previously mentioned (Nobre et al., 2019). In brief, 400 M cells expressing either BNRF1-HA, BOLF1-HA, BPLF1-HA or BLRF2-HA were induced into lytic cycle, harvested and lysed in MCLB buffer (50 mM Tris-HCI pH 7.5, 300 mM NaCl, 0.5% v/v NP40, 1 mM DTT and Roche protease inhibitor cocktail). Samples were tumbled for 15 mins at 4°C and subjected to centrifugation at 16000xg for 15 mins at 4°C. Lysates were then filtered through a 0.7 μm filter and incubated for 3 h with immobilized mouse monoclonal anti-HA agarose resin (Sigma). Duplicates samples were combined and washed seven times with lysis buffer, followed by seven PBS washes. After that, proteins bound to the anti-HA resin were eluted twice by adding 200 μl of 250 μg/mL HA peptide in PBS at 37°C for 30 mins with agitation. Finally, proteins were precipitated with 20% Trichloroacetic acid (TCA), washed once with 10% TCA, washed three times with cold acetone and dried to completion using a centrifugal evaporator. Samples were then resuspended in digestion buffer (50 mM Tris-HCl pH 8.5, 10% acetonitrile (AcN), 1 mM DTT, 10 μg/mL Trypsin (Promega)) and incubated overnight at 37°C with agitation. The reaction was quenched with 50% formic acid (FA), subjected to C18 solid-phase extraction, and vacuum-centrifuged to complete dryness. Samples were reconstituted in 4% AcN/5% FA and divided into technical duplicates prior to LC-MS/MS on an Orbitrap Lumos.

#### LC-MS analysis

Peptides for each sample were analyzed in technical duplicate, with the run order reversed from one batch of replicate analyses to the next to ensure that any carry-over was different in each case. Two washes were used between each sample to further minimize carry-over. Mass spectrometry data were acquired using an Orbitrap Fusion Lumos. An Ultimate 3000 RSLC nano UHPLC equipped with a 300 μm ID x 5 mm Acclaim PepMap μ-Precolumn (Thermo Fisher Scientific) and a 75 μm ID x 75 cm 2 μm particle Acclaim PepMap RSLC analytical column was used. Loading solvent was 0.1% v/v FA, and the analytical solvents were (A) 0.1% v/v FA and (B) 80% v/v AcN + 0.1% v/v FA. All separations were carried out at 55°C. Samples were loaded at 5 μl/min for 5 min in loading solvent before beginning the analytical gradient. The following gradient was used: 3–7% B over 3 min then 7–37% B over 54 min followed by a 4 min wash in 95% B and equilibration in 3% B for 15 min. The following settings were used: MS1, 350–1500 Thompsons (Th), 120,000 resolution, 2 × 105 automatic gain control (AGC) target, 50 ms maximum injection time. MS2, quadrupole isolation at an isolation width of m/z 0.7, higher-energy collisional dissociation (HCD) fragmentation (normalized collision energy (NCE) 34) with fragment ions scanning in the ion trap from m/z 120, 1 × 104 AGC target, 250 ms maximum injection time, with ions accumulated for all parallelizable times. The method excluded undetermined and very high charge states (≥25+). Dynamic exclusion was set to + /− 10 ppm for 25 s. MS2 fragmentation was trigged on precursors 5 × 103 counts and above. Two 45 min washes were included between every affinity purification-mass spectrometry (AP-MS) analysis, to minimize carry-over between samples. 1 μl transport solution (0.1% v/v trifluoroacetic acid) was injected, over the following gradient: 3–40% B over 29 min followed by a 3 min wash at 95% B and equilibration at 3% B for 10 min.

#### CompPASS identification of high confidence protein interactors

To identify interactors for each bait, replicate pairs were combined to attain a summary of proteins identified in both runs. Data reported for each protein in every IP in the dataset include: (a) the number of peptide spectrum matches (PSMs) averaged between technical replicates; (b) an entropy score, which compares the number of PSM between replicates to eliminate proteins that are not detected consistently; (c) a z-score, calculated in comparison to the average and standard deviation of PSMs observed across all IPs; and (d) an NWD score, which reflects (i) how frequently this protein was detected and (ii) whether it was detected reproducibly. NWD scores were calculated as described in (Behrends et al., 2010) using the fraction of runs in which a protein was observed, the observed number of PSMs, the average and standard deviation of PSMs observed for that protein across all IPs, and the number of replicates (1 or 2) containing the protein of interest. Protein interactors identified were filtered with false discovery rate (FDR) <0.02; average peptide spectrum match ≥1.5; entropy score ≥0.75 and top 2% WD/z-score.

### QUANTIFICATION AND STATISTICAL ANALYSIS

Unless otherwise indicated, all bargraphs and linegraphs represent the arithmetic mean of three independent experiments (n = 3), with error bars denoting SEM. Significance between the control and experimental groups, or indicated pairs of groups, was assessed using the unpaired Student’s t test in the GraphPad Prism 7 software. P values correlate with symbols as follows, unless otherwise indicated: ns = not significant, p > 0.05; *p < 0.05; **p < 0.01; ***p < 0.001; ****p < 0.0001. Principal Component Analysis (PCA) of RNAseq datasets were determined and visualized using R 3.3.2. Pathway analysis was performed and visualized by using WebGestalt functional enrichment analysis web tool.

## Supplementary Material

1

## Figures and Tables

**Table:** KEY RESOURCES TABLE

REAGENT or RESOURCE	SOURCE	IDENTIFIER
Antibodies		
Anti-ATRX rabbit monoclonal antibody (D1N2E)	Cell Signaling Technology	#14820; RRID: AB_2798630
Anti-BNRF1 rabbit polyclonal antibody	A gift from Paul Lieberman	N/A
Anti-BMRF1 mouse monoclonal antibody OT14E	A gift from Jaap M Middeldorp	N/A
Anti-EBV ZEBRA mouse monoclonal antibody (BZ1)	Santa Cruz Biotechnology	sc-53904; RRID: AB_783257
Anti-CAPN1 mouse monoclonal antibody	Proteintech Group	67732-1-Ig
Anti-CAPNS1 rabbit polyclonal antibody	Proteintech Group	25057-1-AP; RRID: AB_2879876
Anti-human CD23 Antibody (PE conjugate)	Biolegend	338507; RRID: AB_1279179
Anti-DAXX rabbit monoclonal antibody (25C12)	Cell Signaling Technology	#4533; RRID: AB_2088778
Anti-DDX46 rabbit polyclonal antibody	Proteintech Group	16927-1-AP; RRID: AB_2090927
Anti-GAPDH XP® rabbit monoclonal (D16H11)	Cell Signaling Technology	#5174; RRID:AB_10622025
Anti-gp350 mouse monoclonal antibody OT6	A gift from Jaap M Middeldorp	N/A
Anti-HA.11 tag mouse monoclonal antibody (16B12)	Biolegend	901513; RRID: AB_2820200
Anti-NSMCE2 rabbit polyclonal antibody	Proteintech Group	13627-1-AP; RRID: AB_10637854
Anti-NDNL2(NSMCE3) rabbit polyclonal antibody	Proteintech Group	27488-1-AP; RRID: AB_2880885
Anti-PARP rabbit monoclonal antibody (46D11)	Cell Signaling Technology	#9532; RRID: AB_659884
Anti-DNA-RNA hybrid antibody (S9.6)	Millipore	MABE1095; RRID: AB_2861387
Anti-SMC5 rabbit polyclonal antibody	Proteintech Group	14178-1-AP; RRID: AB_2192775
Anti-SMC6L1 rabbit monoclonal antibody	Boster Biological Technology	A01554-1
Anti-V5-Tag rabbit monoclonal antibody (D3H8Q)	Cell Signaling Technology	#13202; RRID: AB_2687461
Anti-Mouse IgG HRP-coupled secondary antibody	Cell Signaling Technology	#7076; RRID: AB_330924
Anti-Rabbit IgG HRP-coupled secondary antibody	Cell Signaling Technology	#7074; RRID: AB_2099233
Anti-dsRNA mouse monoclonal antibody (clone rJ2)	Millipore	MABE1134; RRID: AB_2819101
Anti-Ubiquitin mouse monoclonal antibody (P4D1)	Cell Signaling Technology	#3936; RRID: AB_331292
Goat anti-Rabbit IgG (H+L) Cross-Adsorbed Secondary Antibody, Alexa Fluor 568	Thermo Fisher Scientific	A-11011; RRID: AB_141416
Goat anti-Mouse IgG (H+L) Cross-Adsorbed Secondary Antibody, Alexa Fluor 488	Thermo Fisher Scientific	A-11001; RRID: AB_143160
Anti-streptavidin, Alexa Fluor 647 conjugate	Thermo Fisher Scientific	S32357
Anti-Human IgG rabbit polyclonal antibody (Gamma-Chains)	Agilent	A042402; RRID: AB_578517
Bacterial and virus strains		
293 cells with the 2089-EBV-BAC (Bacterial Artificial Chromosome) for production of recombinant EBV	Bo Zhao	N/A
Chemicals, peptides, and recombinant proteins		
Pierce™ Protein A/G Magnetic Beads	Thermo Fisher Scientific	88803
Pierce™ Anti-HA Magnetic Beads	Thermo Fisher Scientific	88837
T4 DNA ligase	New England Biolabs	M0202L
Proteinase K	New England Biolabs	P8107S
Doxycycline hyclate	Sigma-Aldrich	D9891-1G
(Z)-4-Hydroxytamoxifen	Sigma-Aldrich	H7904-25MG
Sodium butyrate, >98%, Alfa Aesar™	Thermo Fisher Scientific	AAA1107922
NAE Inhibitor, MLN4924	Sigma-Aldrich	5.05477
Bortezomib (PS-341)	APExBIO	A2614
InSolution™ Leupeptin, Hemisulfate, Microbial	Millipore	509281
E-64 protease inhibitor	Millipore	324890-5MG
Calpeptin ≥98% (HPLC)	Millipore	C8999
5-Ethynyl-2’-deoxycytidine, (EdC)	Sigma-Aldrich	T511307-5MG
Biotin Picolyl Azide	Click Chemistry Tools	1167-25
Cupric Sulfate Pentahydrate	Thermo Fisher Scientific	C489-500
Sodium ascorbate	Sigma-Aldrich	A4034-500G
RNase H	New England Biolabs	M0297L
Phosphonoacetic acid (PAA)	Sigma-Aldrich	284270-10G
Benzonase® Nuclease	Sigma-Aldrich	E1014-5KU
Exonuclease T	New England Biolabs	M0265S
T7 Endonuclease I	New England Biolabs	M0302S
DNase I	Roche	10104159001
Formaldehyde solution	Sigma-Aldrich	F8775
cOmplete™, Mini, EDTA-free Protease Inhibitor Cocktail	Roche	11697498001
Puromycin Dihydrochloride	Thermo Fisher Scientific	A1113803
Hygromycin B	Millipore	400052
G418 Sulfate Solution (50 mg/mL)	GeminiBio	400113
Blasticidin	InvivoGen	ant-bl-5
TransIT®-LT1 Transfection Reagent	Mirus Bio	MIR 2306
NP 40	Sigma-Aldrich	74385-1L
Sequencing Grade Modified Trypsin (Mass Spec Grade) (lyophilized)	Promega	V5111-5x20μg
HA Synthetic Peptide	Thermo Fisher Scientific	26184-5mg
Monoclonal Anti-HA–Agarose antibody produced in mouse	Sigma-Aldrich	A2095-1ML
Critical commercial assays		
RNeasy Mini Kit	Qiagen	74104
Cy5® Conjugation Kit (Fast)	Abcam	Ab188288
QiAquick PCR Purification Kit	Qiagen	28106
Blood & Cell Culture DNA Maxi Kit	Qiagen	13362
QIAprep Spin Miniprep Kit	Qiagen	27106
DNeasy Blood& Tissue Kit	Qiagen	69504
QIAquick Gel Extraction Kit	Qiagen	28704
RNase-Free DNase Set	Qiagen	79254
iScript Reverse Transcription Supermix for RT-qPCR	BIO-RAD	1708841
Power SYBR Green PCR Master Mix	Applied Biosystems	4367659
Gateway™ LR Clonase™ II Enzyme Mix	Invitrogen	11789-020
NEBNext® Poly(A) mRNA Magnetic Isolation Module	New England Biolabs	E7490S
NEBNext® Ultra™ II Directional RNA Library Prep with Sample Purification Beads	New England Biolabs	E7765S
NEBNext® Multiplex Oligos for Illumina® (Index Primers Set 2)	New England Biolabs	E7500S
NEBNext® Multiplex Oligos for Illumina® (Index Primers Set 1)	New England Biolabs	E7335S
EasySep™ Human T Cell Isolation Kit	Stemcell Technologies	17954
RosetteSep™ Human Monocyte Enrichment Cocktail	Stemcell Technologies	15064
Deposited data		
RNAseq	This paper	GEO: GSE182349
Mendeley dataset	This paper	Mendeley Data: https://doi.org/10.17632/5v545cw8t7.1
Experimental models: Cell lines		
EBV + Burkitt lymphoma P3HR-1 ZHT/RHT	A gift from Eric Johannsen and Elliott Kieff	N/A
EBV+ Burkitt lymphoma Akata-Cas9	Guo et al. (2020)	N/A
EBV+ Burkitt lymphoma Daudi-Cas9	(Ma et al., 2017)	N/A
HEK293T	ATCC	CRL-3216
EBV+ Gastric carcinoma AGSiZ	A gift from Sankar Swaminathan	N/A
EBV+ Burkitt lymphoma Raji	ATCC	ATCC® CCL-86™
EBV- Burkitt lymphoma BJAB-Cas9	A gift from Bo Zhao	N/A
Oligonucleotides		
sgRNAs were listed in Table S1	This paper	N/A
Primers for BNRF1 cDNA rescue were listed in Table S1	This paper	N/A
Mutagenesis primers were listed in Table S1	This paper	N/A
qPCR primers for EBV copy number quantification were listed in Table S1	This paper	N/A
ChIP-qPCR primers were listed in Table S1	This paper	N/A
Recombinant DNA		
pLentiGuide-Puro	A gift from Feng Zhang (Sanjana et al., 2014)	Addgene_52963
pLenti SpBsmBI sgRNA Hygro	A gift from Rene Maehr (Pham et al., 2016)	Addgene_62205
pLX-TRC313	Broad Institute	N/A
pLX-402	Broad Institute	N/A
pENTR-BNRF1	Eric Johannsen	N/A
pENTR-BRLF1	Eric Johannsen	N/A
pENTR-BLLF3	Eric Johannsen	N/A
pENTR-BGLF4	Eric Johannsen	N/A
pENTR-GFP	Eric Johannsen	N/A
pENTR-BOLF1	Eric Johannsen	N/A
pENTR-BPLF1	Eric Johannsen	N/A
pENTR-BLRF2	Eric Johannsen	N/A
pENTR-SMC6	DNASU	HsCD00080486
pLX-402-BNRF1	This paper	N/A
pLX-TRC313-BNRF1	This paper	N/A
pLX-402-BRLF1	This paper	N/A
pLX-402-BLLF3	This paper	N/A
pLX-402-BGLF4	This paper	N/A
pLX-402-GFP	This paper	N/A
pLX-402-BOLF1	This paper	N/A
pLX-402-BPLF1	This paper	N/A
pLX-402-BLRF2	This paper	N/A
pLX-402-SMC6	This paper	N/A
Software and algorithms		
Salmon v0.8.2	Patro et al. (2017)	https://combine-lab.github.io/salmon/
DESeq2 V1.18.1	Love et al. (2014)	https://bioconductor.org/packages/release/bioc/html/DESeq2.html
WebGestalt (WEB-based Gene SeT AnaLysis Toolkit)	Liao et al. (2019)	http://www.webgestalt.org/
GraphPad Prism 7	GraphPad Software	https://www.graphpad.com/scientific-software/prism/
Flowjo X	Flowjo LLC.	https://www.flowjo.com/
Biorender	Biorender	https://biorender.com/
ImageJ	ImageJ	https://imagej.nih.gov/ij/
ImageJ- Particle Analyzer	ImageJ	https://imagej.net/imaging/particle-analysis
ImageJ- Interactive 3D Surface Plot	Kai Uwe Barthel	https://imagej.nih.gov/ij/plugins/surface-plot-3d.html
Zeiss Zen Lite (Blue)	Zeiss	https://www.zeiss.com/microscopy/int/products/microscope-software/zen-lite.html
Arivis Vision4D	Arivis	https://imaging.arivis.com/en/imaging-science/arivis-vision4d
Other		
Standard Fetal Bovine Serum, Qualified, USDA-Approved Regions	Thermo Fisher Scientific	10437028
RPMI 1640 Medium	Life Technologies	11875085
DMEM, high glucose, pyruvate	Life Technologies	11995081
Ham’s F-12 Nutrient Mix, GlutaMAX™ Supplement	Thermo Fisher Scientific	31765035

## Highlights

- EBV BNRF1 subverts SMC5/6 cohesin complexes in newly infected and lytic cells
- BNRF1 targets SMC5/6 in a calpain-, Cul7-, and ubiquitin proteasome-dependent pathway
- SMC5/6 restricts EBV replication compartment formation and lytic DNA encapsidation
- SMC5/6 complex interacts with R-loops, including at the EBV origin of lytic replication
