# Supplementary material for: Epstein-Barr virus BNRF1 destabilizes SMC5/6 cohesin complexes to evade its restriction of replication compartments
Source: Cell Rep. Author manuscript; Available in PMC 2022 Apr 5. (PMC8981113; doi:10.1016/j.celrep.2022.110411)
Supplement: 1 [file NIHMS1787420-supplement-1.pdf]

Cell Reports, Volume *38*

Supplemental information

Epstein-Barr virus BNRF1 destabilizes SMC5/6 cohesin complexes to evade its restriction of replication compartments

Stephanie Pei Tung Yiu, Rui Guo, Cassie Zerbe, Michael P. Weekes, and Benjamin E. Gewurz

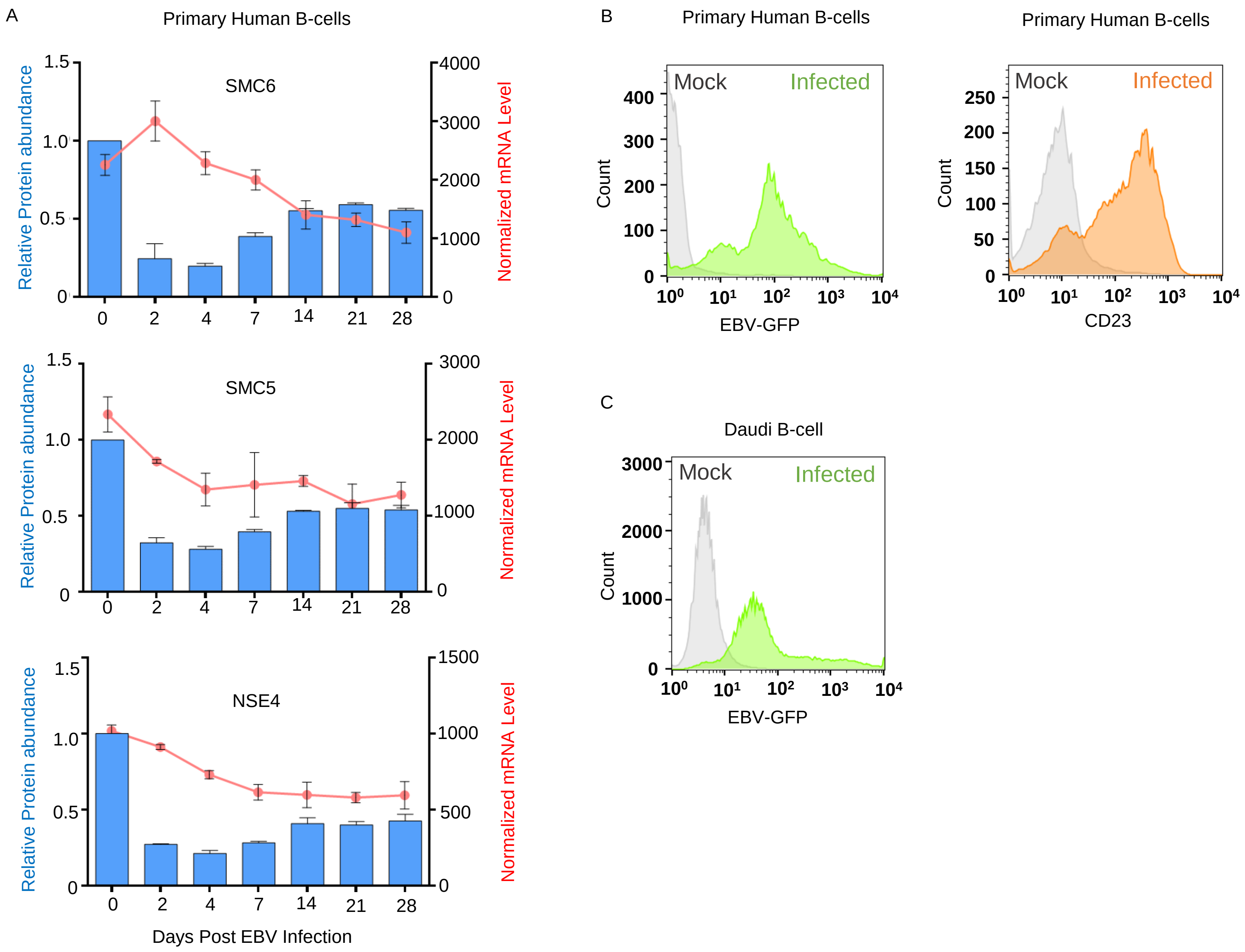
A
Primary Human B-cells
SMC6
SMC5
NSE4
Relative Protein abundance
Normalized mRNA Level
Days Post EBV Infection
B
Primary Human B-cells
Mock
Infected
Count
EBV-GFP
CD23
C
Daudi B-cell
Mock
Infected
Count
EBV-GFP

Figure S2

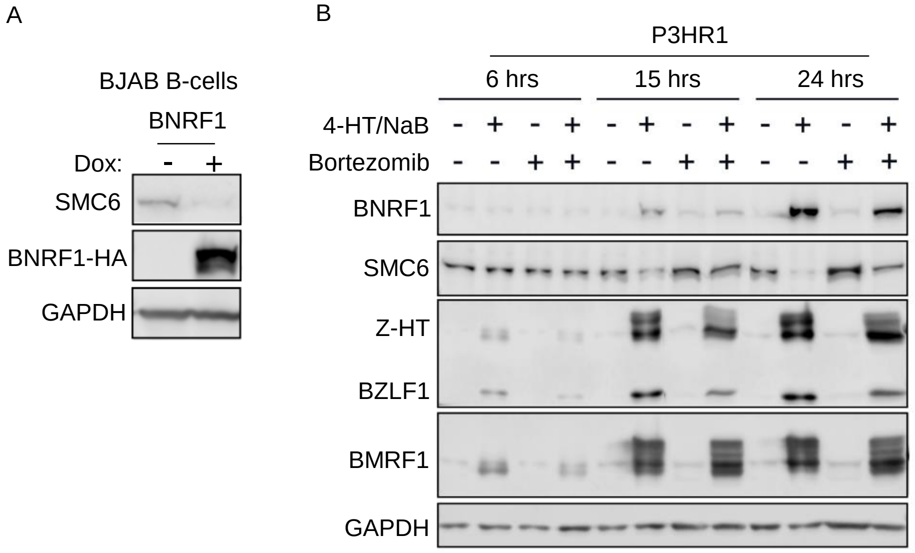

C

Bait	Prey	Z-score
BNRF1	KTN1	2.67
	CAPNS1	2.67
	DAXX	2.66
	ICAL	2.64
	CAPN1	2.61
	RD23A	2.61
	PKRDC	1.53
	MAP4	1.36
	NUCL	1.10
	HNRF1	1.03
	CLH1	0.97
	TFG	0.81

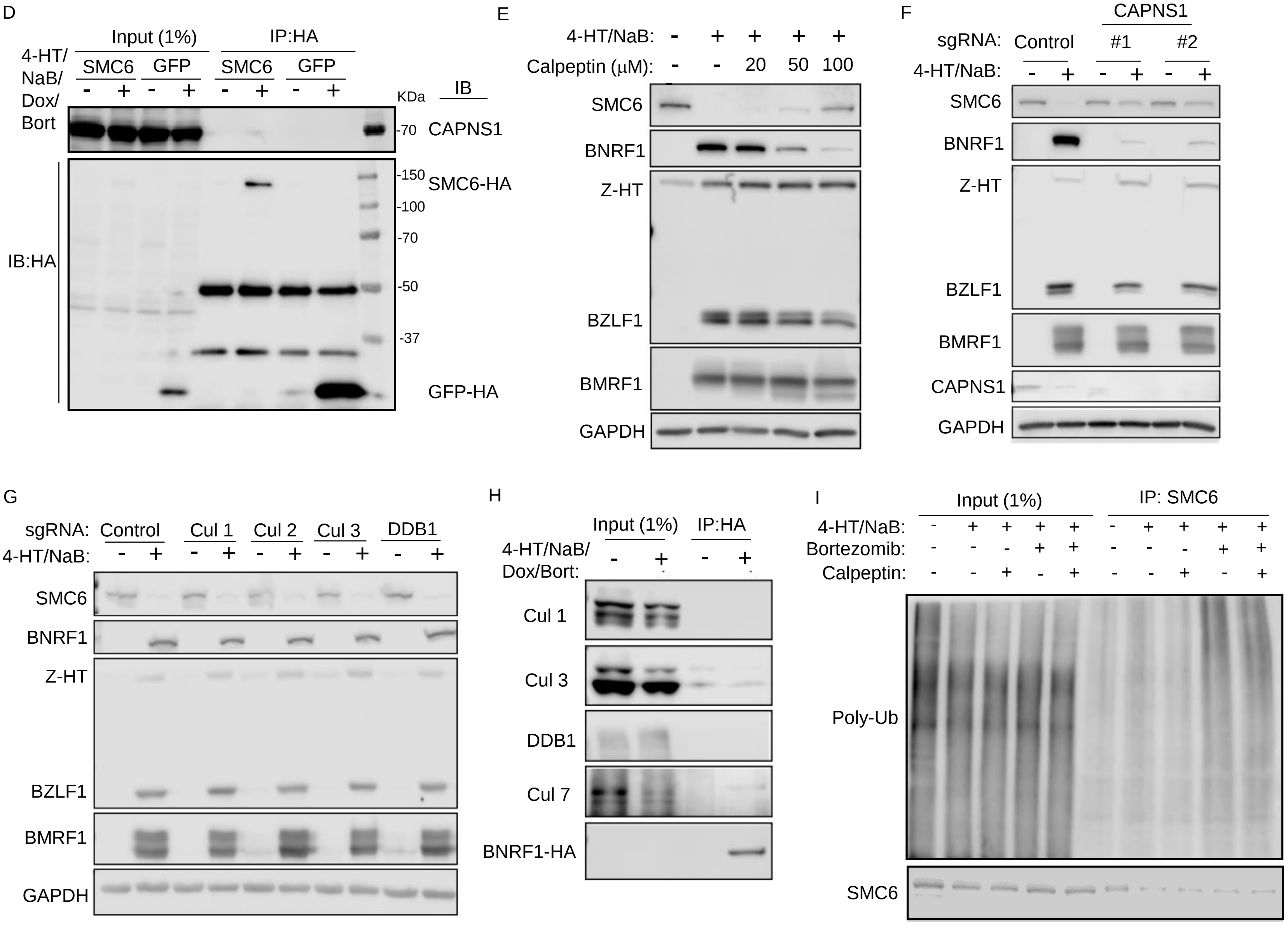

Figure S3

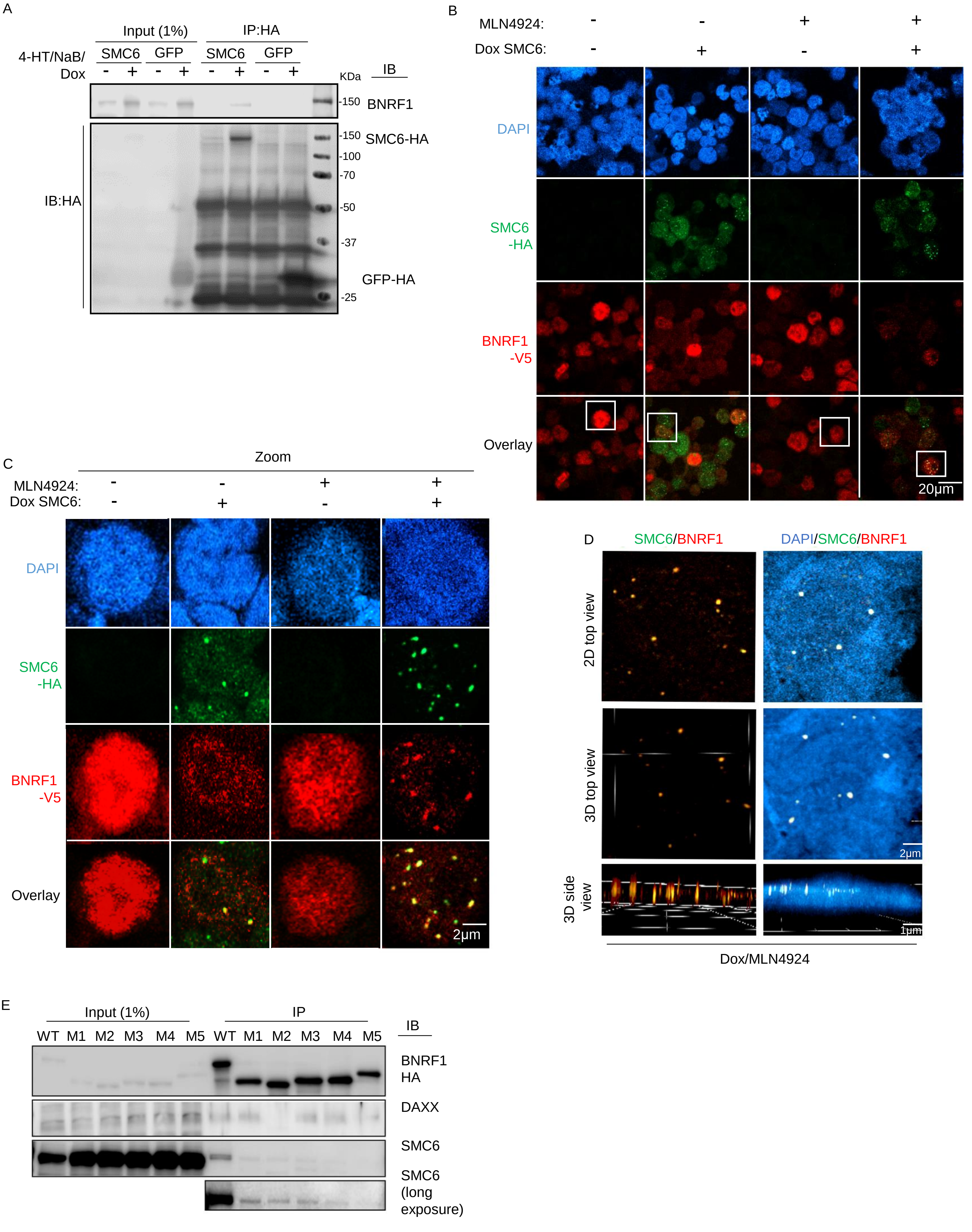
A
Input (1%)
IP:HA
4-HT/NaB/
Dox
SMC6
GFP
SMC6
GFP
KDa
IB
-150
BNRF1
-150
SMC6-HA
-100
-70
-50
-37
-25
IB:HA
GFP-HA
B
MLN4924:
Dox SMC6:
DAPI
SMC6
-HA
BNRF1
-V5
Overlay
20μm
C
Zoom
MLN4924:
Dox SMC6:
DAPI
SMC6
-HA
BNRF1
-V5
Overlay
2μm
D
SMC6/BNRF1
DAPI/SMC6/BNRF1
2D top view
3D top view
3D side view
2μm
1μm
Dox/MLN4924
E
Input (1%)
IP
WT M1 M2 M3 M4 M5 WT M1 M2 M3 M4 M5
IB
BNRF1
HA
DAXX
SMC6
SMC6
(long
exposure)

Figure S4

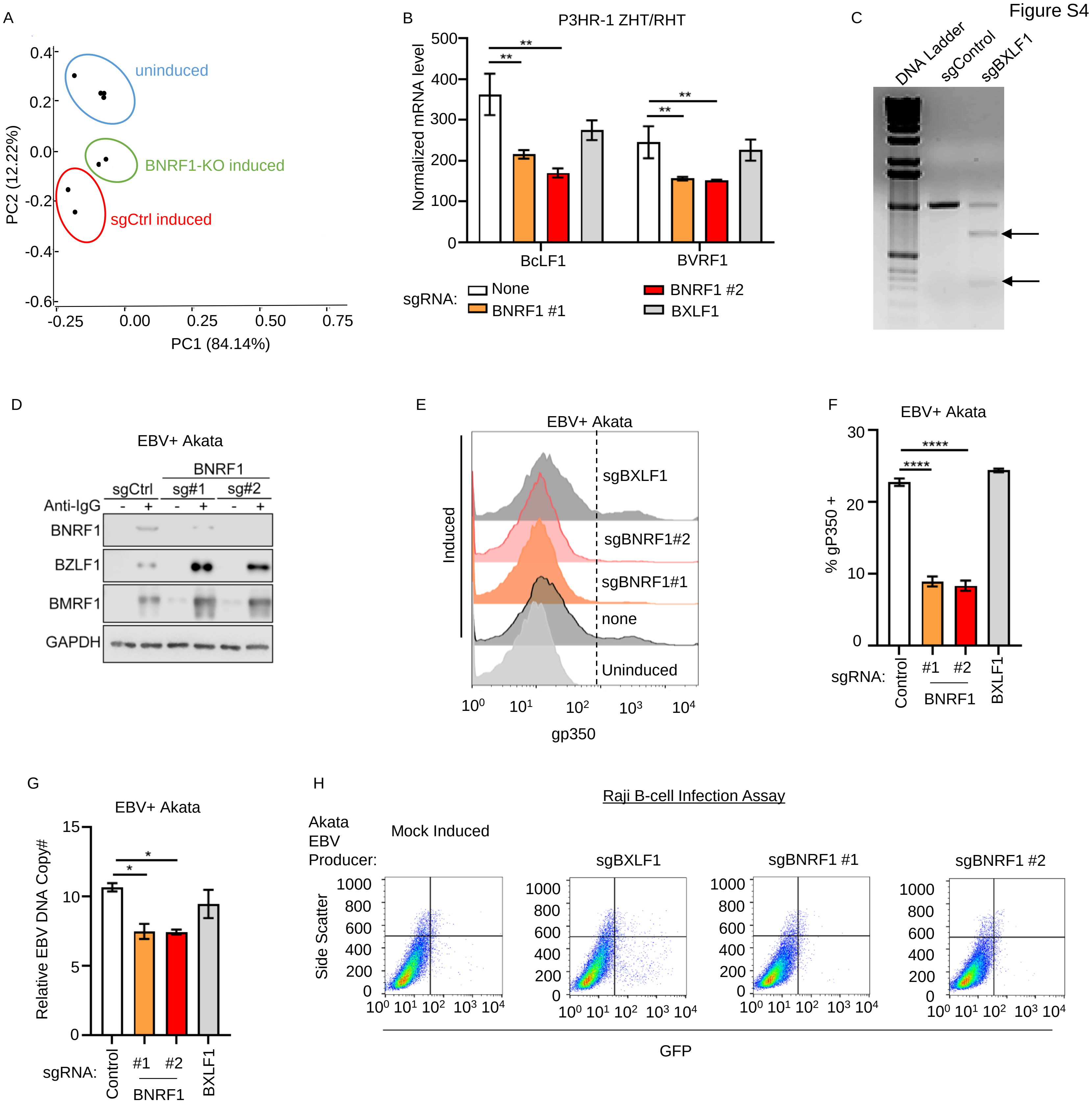
A
uninduced
BNRF1-KO induced
sgCtrl induced
PC2 (12.22%)
PC1 (84.14%)
B
P3HR-1 ZHT/RHT
Normalized mRNA level
BcLF1
BVRF1
sgRNA:
None
BNRF1 #1
BNRF1 #2
BXLF1
C
DNA Ladder
sgControl
sgBXLF1
D
EBV+ Akata
BNRF1
sgCtrl
sg#1
sg#2
Anti-IgG
BNRF1
BZLF1
BMRF1
GAPDH
E
EBV+ Akata
Induced
sgBXLF1
sgBNRF1#2
sgBNRF1#1
none
Uninduced
gp350
F
EBV+ Akata
% gP350 +
sgRNA:
Control
#1
#2
BNRF1
BXLF1
G
EBV+ Akata
Relative EBV DNA Copy#
sgRNA:
Control
#1
#2
BNRF1
BXLF1
H
Raji B-cell Infection Assay
Akata EBV Producer:
Mock Induced
sgBXLF1
sgBNRF1 #1
sgBNRF1 #2
Side Scatter
GFP

Figure S5

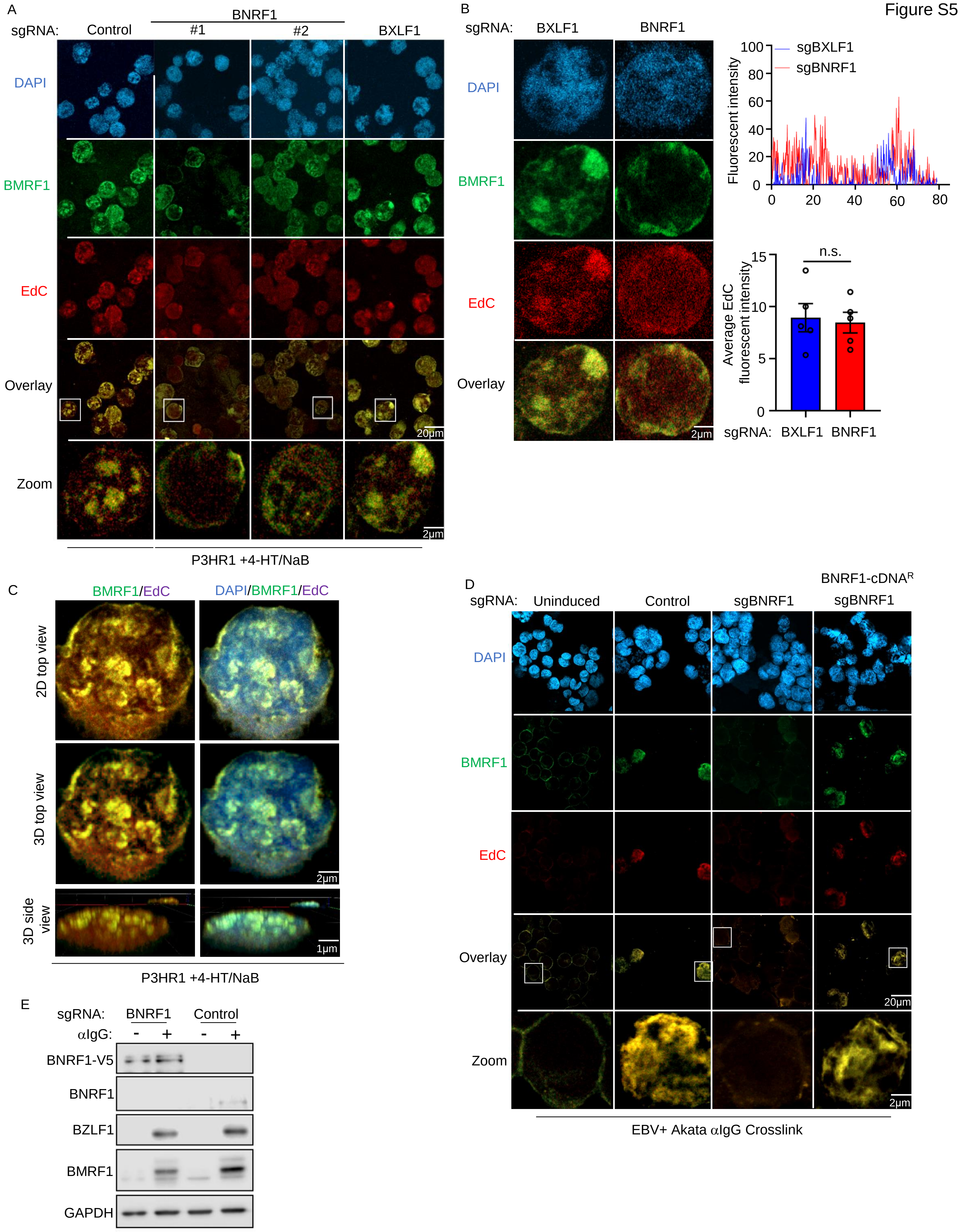

A
sgRNA: Control
BNRF1
#1
#2
BXLF1
DAPI
BMRF1
EdC
Overlay
Zoom
20μm
2μm
P3HR1 +4-HT/NaB
B
sgRNA: BXLF1 BNRF1
DAPI
BMRF1
EdC
Overlay
2μm
Fluorescent intensity
sgBXLF1
sgBNRF1
100
80
60
40
20
0
0 20 40 60 80
Average EdC fluorescent intensity
n.s.
15
10
5
0
sgRNA: BXLF1 BNRF1
C
BMRF1/EdC
DAPI/BMRF1/EdC
2D top view
3D top view
3D side view
2μm
1μm
P3HR1 +4-HT/NaB
D
BNRF1-cDNAR
sgRNA: Uninduced Control sgBNRF1 sgBNRF1
DAPI
BMRF1
EdC
Overlay
Zoom
20μm
2μm
EBV+ Akata αIgG Crosslink
E
sgRNA: BNRF1 Control
αIgG: - + - +
BNRF1-V5
BNRF1
BZLF1
BMRF1
GAPDH

Figure S6

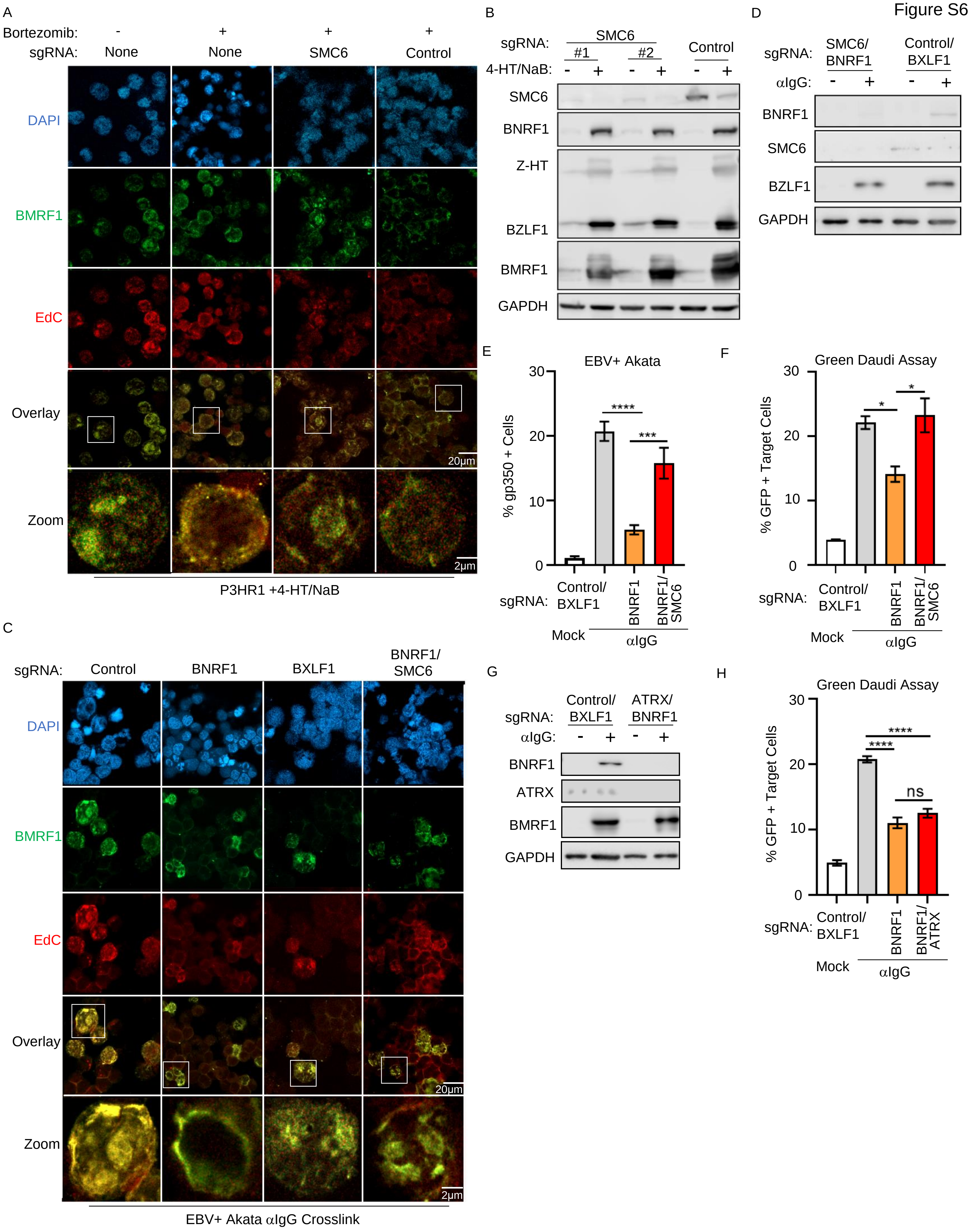

A
Bortezomib: - + + +
sgRNA: None None SMC6 Control
DAPI
BMRF1
EdC
Overlay
Zoom
20μm
2μm
P3HR1 +4-HT/NaB
B
sgRNA: SMC6 #1 #2 Control
4-HT/NaB: - + - + - +
SMC6
BNRF1
Z-HT
BZLF1
BMRF1
GAPDH
D
sgRNA: SMC6/ BNRF1 Control/ BXLF1
αIgG: - + - +
BNRF1
SMC6
BZLF1
GAPDH
E
EBV+ Akata
% gp350 + Cells
30
20
10
0
****
***
sgRNA: Control/ BXLF1 BNRF1 BNRF1/ SMC6
Mock αIgG
F
Green Daudi Assay
% GFP + Target Cells
30
20
10
0
*
*
sgRNA: Control/ BXLF1 BNRF1 BNRF1/ SMC6
Mock αIgG
C
sgRNA: Control BNRF1 BXLF1 BNRF1/ SMC6
DAPI
BMRF1
EdC
Overlay
Zoom
20μm
2μm
EBV+ Akata αIgG Crosslink
G
sgRNA: Control/ BXLF1 ATRX/ BNRF1
αIgG: - + - +
BNRF1
ATRX
BMRF1
GAPDH
H
Green Daudi Assay
% GFP + Target Cells
30
20
10
0
****
****
ns
sgRNA: Control/ BXLF1 BNRF1 BNRF1/ ATRX
Mock αIgG

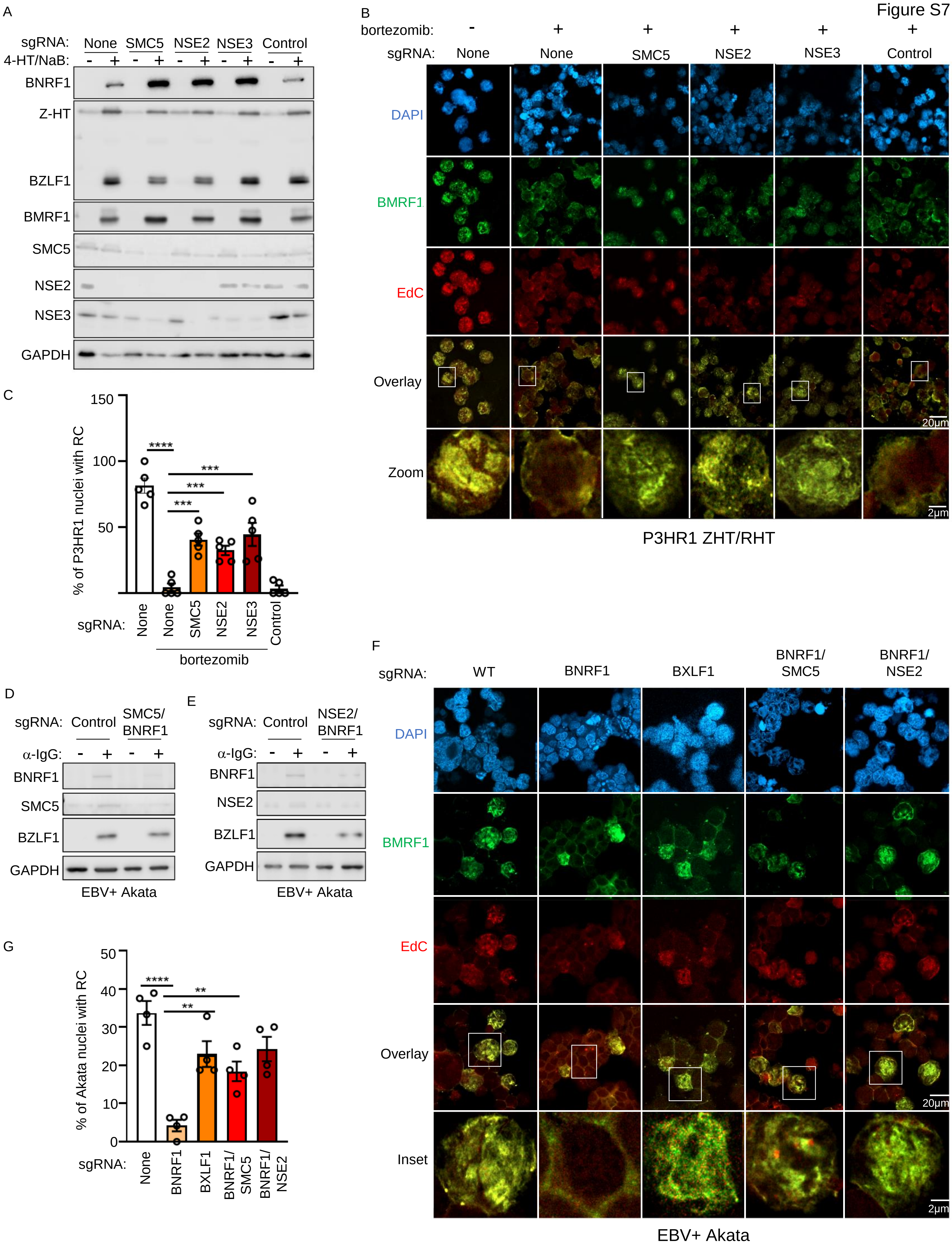

Figure S7
A
sgRNA: None SMC5 NSE2 NSE3 Control
4-HT/NaB: - + - + - + - + - +
BNRF1
Z-HT
BZLF1
BMRF1
SMC5
NSE2
NSE3
GAPDH
B
bortezomib: - + + + + +
sgRNA: None None SMC5 NSE2 NSE3 Control
DAPI
BMRF1
EdC
Overlay
Zoom
20µm
2µm
P3HR1 ZHT/RHT
C
% of P3HR1 nuclei with RC
150
100
50
****
***
***
***
sgRNA: None None SMC5 NSE2 NSE3 Control
bortezomib
D
sgRNA: Control SMC5/ BNRF1
α-IgG: - + - +
BNRF1
SMC5
BZLF1
GAPDH
EBV+ Akata
E
sgRNA: Control NSE2/ BNRF1
α-IgG: - + - +
BNRF1
NSE2
BZLF1
GAPDH
EBV+ Akata
F
sgRNA: WT BNRF1 BXLF1 BNRF1/ SMC5 BNRF1/ NSE2
DAPI
BMRF1
EdC
Overlay
Inset
20µm
2µm
EBV+ Akata
G
% of Akata nuclei with RC
50
40
30
20
10
0
****
**
**
sgRNA: None BNRF1 BXLF1 BNRF1/ SMC5 BNRF1/ NSE2

Supplementary Figures:

Figure S1. Related to Figure 1: the SMC5/6 cohesin complex is depleted by incoming EBV.

(A) Relative protein abundances (blue) and normalized mRNA levels (red) of SMC6, SMC5 and NSE4 at the indicated days post infection of primary human B-cells. SEM values from n=4 proteomic and n=3 RNA-seq replicates are shown. Data are from (Ersing et al., 2017; Wang et al., 2019a).

(B) FACS analysis of GFP levels (left) and CD23 levels (right) in primary human B-cells mock infected or infected by Akata GFP+ EBV at 48 h post-infection (hpi). GFP and CD23 upregulation were used as markers of EBV infection.

(C) FACS analysis of GFP levels in Daudi cells super-infected by Akata GFP+ EBV at 48 hpi.

Figure S2. Related to Figure 2: BNRF1 targets SMC5/6 for proteasomal degradation in a calpain-and cullin-dependent manner.

(A) WCL of EBV- BJAB B-cells mock or doxycycline (5 µg/ml) induced for BNRF1 expression for 24 h.

(B) Immunoblot analysis of WCL from P3HR-1 ZHT/RHT cells induced for lytic replication and treated with bortezomib as indicated.

(C) Table of high confidence host proteins interactors that selectively co-purified with HA-BNRF1, as in Figure 2F.

(D) Immunoblot analysis of 1% input and anti-HA immunopurified GFP or SMC6 complexes from P3HR-1 ZHT/RHT untreated or treated with doxycycline (5 µg/ml) and bortezomib (5 nM) for 6 h, as indicated. Representative of n=2 independent experiments.

(E) Immunoblot analysis of WCL from P3HR-1 ZHT/RHT cells induced for lytic replication in the presence of calpeptin for 24 h, as indicated.

(F) Immunoblot analysis of WCL from P3HR-1 ZHT/RHT expressing the indicated sgRNAs and induced for lytic replication for 24 h, as indicated.

(G) Immunoblot analysis of WCL from P3HR-1 ZHT/RHT cells expressing the indicated sgRNAs and induced for lytic replication, as indicated.

(H) Immunoblot analysis of 1% input and anti-HA immunopurified BNRF1 from P3HR-1 ZHT/RHT treated with doxycycline (5 µg/ml), bortezomib (5 nM), 4-HT and NaB for 6 h, as indicated. Representative of two independent experiments.

(I) Immunoblot analysis of 1% input and SMC6 complexes immunopurified from P3HR-1 ZHT/RHT that were induced into lytic cycle by 4-HT/NaB and treated with bortezomib (5 nM) and/or calpeptin (100 µM), as indicated. Samples were blotted for poly-ubiquitin (poly-Ub) using the antibody P4D1 or for SMC6. Representative of two independent experiments.

Figure S3. Related to Figure 3: BNRF1 associates with SMC6 in nuclear puncta.

(A) Immunoblot analysis of 1% input and anti-HA immunopurified GFP or BNRF1 complexes from P3HR-1 ZHT/RHT untreated or treated with doxycycline (5 µg/ml) to induce cDNA, bortezomib (5 nM) for 6 h, as indicated. Representative of two independent experiments.

(B) Immunofluorescence analysis of DAPI, doxycycline-induced HA-tagged SMC6 and stably expressed V5-tagged BNRF1 in EBV+ Akata. Cells were treated with doxycycline (5 µg/ml) and bortezomib (5 nM) for 12 h. Representative of n=3 experiments.

(C) Zoom image of cells from S3B boxed in white.

(D) 3D reconstruction of cells as in (B). Representative of three independent experiments.

(E) Immunoblot analysis of 1% input and anti-HA immunopurified complexes from P3HR-1 ZHT/RHT that were doxycycline (5 µg/ml) induced for wildtype (WT) or deletion mutant BNRF1 constructs in the presence of bortezomib (5 nM) for 6 h. Representative of n=2 experiments.

Figure S4. Related to Figure 4: BNRF1 supports late lytic cycle progression.

(A) RNA-seq dataset principal component (PC) analysis, as in Figure 4A, of P3HR-1 ZHT/RHT cells expressing control or BNRF1 sgRNAs and uninduced or induced, as indicated.

(B) Mean ±SEM values from n=3 replicates of qRT-PCR analysis of late gene BcLF1 and BCRF1 transcripts from P3HR-1 ZHT/RHT cells with BXLF1 or BNRF1 sgRNAs induced by 4-HT/NaB for 24 h.

(C) T7-endonuclease DNA mismatch assay of P3HR-1 ZHT/RHT cells with control or BXLF1 sgRNAs. Arrows indicating mismatch cleavage products, indicative of successful EBV genomic CRISPR editing.

(D) Immunoblot analysis of WCL from EBV+ Akata cells with control or BNRF1 sgRNAs, induced into lytic cycle with anti-human IgG (15 µg/ml) as indicated, for 48 h, representative of n=3 replicates.

(E) FACS plot of PM gp350 levels in EBV+ Akata cells with control BXLF1 or BNRF1 sgRNAs, induced by anti-human IgG (15 µg/ml) as indicated, for 48 h.

(F) Mean ±SEM PM gp350 from n=5 replicates, as in E.

(G) Mean ±SEM qRT-PCR values from n=3 replicates of EBV intracellular genome copy number from EBV+ Akata cells with BXLF1 or BNRF1 sgRNA induced by anti-human IgG (15 µg/ml) for 48 h.

(H) FACS plots of green Raji assay analysis of infectious EBV titers produced from EBV+ Akata with BXLF1 or BNRF1 sgRNA induced by anti-human IgG (15 µg/ml) for 48 h and then co-incubated with Raji cells. ****p < 0.0001. ***p<0.001. **p < 0.01.

Figure S5. Related to Figure 5: BNRF1 is critical for viral replication compartment formation.

(A) Confocal immunofluorescence analysis of P3HR-1 ZHT/RHT cells with the indicated sgRNA induced for 24 h with 4-HT/NaB and stained for DAPI, BMRF1 or EdC. Zoomed images of cells in white boxes are shown below. Representative of n=3 replicates.

(B) Total fluorescent intensity analysis in 5 randomly selected images of cells with control BXLF1 or BNRF1 sgRNA expression, a representative panel of which is shown on the left. Shown bottom right are the average EdC fluorescence intensity ± SEM values from 5 fields.

(C) 3D reconstruction of P3HR1 ZHT/RHT control cells induced by 4-HT/NaB as in (A). Side view: x- (red), y- (green), z- (blue) coordinates.

(D) Confocal immunofluorescence analysis of EBV+ Akata cells expressing the indicated sgRNA and BNRF1 rescue cDNA that were induced by anti-human IgG (15 µg/ml) for 48 h and stained for DAPI, BMRF1 or EdC. Zoomed images of cells in white boxes are shown below. Representative of n=3 replicates.

(E) Immunoblot analysis of WCL from EBV+ Akata cells with control or BNRF1 sgRNAs induced by anti-human IgG (15 µg/ml) for 48 h. Representative of n=2 replicates.

Figure S6. Related to Figure 5: BNRF1 counteracts RC suppression by SMC6.

(A) Confocal immunofluorescence analysis of P3HR-1 ZHT/RHT cells with the indicated sgRNA induced for 24 h with 4-HT/NaB and stained for DAPI, BMRF1 or EdC. Zoomed images of cells in white boxes are shown below. Representative of n=3 replicates.

(B) Immunoblot analysis of WCL from P3HR-1 ZHT/RHT cells with the indicated sgRNAs and induced for lytic replication, as indicated. Representative of n=3 replicates.

(C) Confocal immunofluorescence analysis of EBV+ Akata cells with the indicated sgRNA induced for 48 h by anti-human IgG (15 µg/ml) and stained for DAPI, BMRF1 or EdC. Zoomed images of cells in white boxes are shown below. Representative of n=3 replicates.

(D) Immunoblot analysis of WCL from EBV+ Akata cells with the indicated sgRNAs and induced for lytic replication, as indicated. Representative of n=3 replicates.

(E) Mean ±SEM values of FACS analysis of % gp350+ cells from n=3 replicates of EBV+ Akata cells expressing the indicated sgRNAs and mock induced or induced for lytic replication, as indicated.

(F) Mean ±SEM values of infectious EBV titers produced by EBV+ Akata with the indicated sgRNAs and induced by anti-human IgG (15 µg/ml) for 48 h. Data are from n=3 green Daudi assay replicates. *$p<0.05$.

(G) Immunoblot analysis of WCL from EBV+ Akata cells with the indicated sgRNAs and induced for lytic replication, as indicated. Representative of n=2 replicates.

(H) Mean ±SEM values of infectious EBV titers produced by EBV+ Akata with the indicated sgRNAs and induced by anti-human IgG (15 µg/ml) for 48 h. Data are from n=3 green Daudi assay replicates. ****$p < 0.0001$, ns=non-significant.

Figure S7. Related to Figure 5: multiple SMC5/6 complex components are important for RC suppression in absence of BNRF1.

(A) Immunoblot analysis of WCL from P3HR-1 ZHT/RHT with the indicated sgRNA and induced by 4-HT/NaB for 24 h, as indicated. Representative of two independent experiments.

(B) Confocal immunofluorescence analysis of P3HR-1 ZHT/RHT cells with the indicated sgRNAs induced by 4HT/NaB for 24 h and treated with bortezomib (5 nM), as indicated.

(C) Mean ±SEM values of percentages of nuclei with RC from n=3 replicates, as in B, using data from 5 randomly selected panels of 75 nuclei, using the ImageJ “Particle Analysis” plugin.

(D) Immunoblot analysis of WCL from EBV+ Akata cells with control or BNRF1/SMC5 sgRNAs, as indicated, induced by anti-human IgG (15 µg/ml) for 48 h.

(E) Immunoblot analysis of WCL from EBV+ Akata cells with control or BNRF1/NSE2 sgRNAs, as indicated, induced by anti-human IgG (15 µg/ml) for 48 h.

(F) Confocal immunofluorescence analysis of EBV+ Akata cells with the indicated sgRNAs induced by anti-human IgG (15 µg/ml) for 48 h.

(G) Mean ±SEM values of percentages of nuclei with RC from n=3 replicates, as in F, using data from 4 randomly selected panels of 80 nuclei, using the ImageJ “Particle Analysis” plugin.

Blots and images are representative of n=2 replicates. ****p < 0.0001, ***p < 0.001, **p<0.01.

Supplementary Table	
CRIPSR analysis	
BNRF1 sg#1 (antisense) _Forward	5’ – CGA GTA AGT GTC TCG CAG CG– 3’
BNRF1 sg#2_Forward	5’ – CTC CAC GCG AAG CAC GTA CG – 3’
BXLF1_Forward	5’ – TTG TAG TCC CTG AAC CGA TG – 3’
SMC5 sg#1_Forward	5’ – TTT ATT TCT CTC ATA CCT GA – 3’
SMC5 sg#2_Forward	5’ – CTG CAA CAG CGG CAG CTG CG – 3’
SMC6 sg#1_Forward	5’ – AAT AGC CTA ATT GAC ATG AG – 3’
SMC6 sg#2_Forward	5’ – TTT CTT ATA ACT AGG CTC CG – 3’
NSMCE2 sg#1_Forward	5’ – ATA TAG TAT GGA CAA GGC AA – 3’
NSMCE2 sg#2_Forward	5’ – GCA ACT AAA CCA TTA TGT AA – 3’
NSMCE3 sg#1_Forward	5’ – GAG ACA TGT TGC AAA AAC CG – 3’
NSMCE3 sg#2_Forward	5’ – GAG CCA TAG CGG AAA CCC CG – 3’
CAPNS1 sg#1_Forward	5’ – TCA CAG GCG GGG TTA CCG AG – 3’
CAPNS1 sg#2_Forward	5’ – CTG CAC CGA GTG GTT CCG CA – 3’
cDNA Rescue	

Genomic DNA	5' – CGA GTA AGT GTC TCG CAG CGC GGA – 3'
Rescue cDNA_Forward	5' – CGA GTA AGT GTC TCG CAG CGC AGA – 3'
Rescue cDNA sequence surrounding the PAM (underlined) mutation site (in red); sgRNA sequence in bold	5' – TGAAGGACCAAGTGGCC**CGAGTAAGTGTCTCGCA GCG**CAGACACGATCTTTAGCTCGTCGGC – 3'
BNRF1 mutagenesis	
BNRF1 M1 del3-300aa_Forward	5' – GGG GAC AAC TTT GTA CAA AAA AGT TGG CAC CAT GGA AGT TAA TGC AAT AGC ATC ATC G – 3'
BNRF1 M2 del301-600aa_Forward	5' – CAC CCC GGC CTC TTT CCC TTC TCT CCG TCT TAC GAG TTG CCC TG – 3'
BNRF1 M3 del601-900aa_Forward	5' – ATG TGG ACG AGA GCA TGG ACA TCC AGC GGG GAG TGA CCA TCA C – 3'
BNRF1 M4 del901-1200aa_Forward	5' – GTG GAG ATG GCC CTG GCC GGG CTG CCT TGT TGG GTG CAA GGC TC – 3'
BNRF1 M5 del1101-1312aa_Forward	5' – GGG GAC AAC TTT GTA CAA AAA AGT TGG CAC CAT GGA AGA GAG GGG CAG G – 3'
qPCR primer sequence	
BALF5_Forward	5' – GAG CGA TCT TGG CAA TCT CT – 3'
BALF5_Reverse	5' – TGG TCA TGG ATC TGC TAA ACC – 3'
ChIP-qPCR primer sequence	
OrilytR_Forward	5' – CGC TGG TTA AGC TGA CGA CCT – 3'
OrilytR_Reverse	5' – GCC CTG GCT AGG AAA GGG AGG AA – 3'
